# Nineteenth-century land use shapes the current occurrence of some plant species, but weakly affects the richness and total composition of Central European grasslands

**DOI:** 10.1007/s10980-024-02016-6

**Published:** 2025-01-13

**Authors:** Gabriele Midolo, Hana Skokanová, Adam Thomas Clark, Marie Vymazalová, Milan Chytrý, Stefan Dullinger, Franz Essl, Jozef Šibík, Petr Keil

**Affiliations:** 1 Department of Spatial Sciences, Faculty of Environmental Sciences, Czech University of Life Sciences Prague, Kamýcká 129, 165 00 Praha - Suchdol, Czech Republic; 2 Department of Landscape Ecology, Silva Tarouca Research Institute for Landscape and Ornamental Gardening, Brno, Czech Republic; 3 Department of Biology, University of Graz, Graz, Austria; 4 Department of Botany and Zoology, Faculty of Science, Masaryk University, Brno, Czech Republic; 5 Division of Biodiversity Dynamics and Conservation, Department of Botany and Biodiversity Research, University Vienna, Vienna, Austria; 6 Division of BioInvasions, Global Change & Macroecology, Department of Botany and Biodiversity Research, University of Vienna, Vienna, Austria; 7 Plant Science and Biodiversity Center, Slovak Academy of Sciences, Bratislava, Slovakia

**Keywords:** Bioindication, Disturbance, Ellenberg indicator values, European history, Hemeroby, Historical landscape, Vascular plant

## Abstract

**Context:**

Historical land use is thought to have influenced plant community diversity, composition and function through the local persistence of taxa that reflect ecological conditions of the past.

**Objectives:**

We tested for the effects of historical land use on contemporary plant species richness, composition, and ecological preferences in the grassland vegetation of Central Europe.

**Methods:**

We analyzed 6975 vegetation plots sampled between 1946 and 2021 in dry, mesic, and wet grasslands in the borderland between Austria, the Czech Republic, and Slovakia. Using 1819–1853 military maps, we assigned each plot to a historical land-use category (arable land, forest, grassland, settlement, permanent crop, and water body). We modeled the response of species richness, composition, and plant ecological preferences to the historical land use including contemporary covariates.

**Results:**

Nineteenth-century land use explained little overall variation in species richness and composition, whereas more variation was explained by contemporary environmental conditions. However, we found that ecological preferences of some species were associated with specific historical land uses. Specifically, species more frequently occurring in historically forested grasslands showed lower light and disturbance frequency indicator values, while those associated with former settlements displayed higher disturbance severity indicator values.

**Conclusions:**

We conclude that signatures of specific land-use conversions, including the restoration of grasslands in human-impacted areas, may still be detectable in grasslands even 200 years into the future. However, while local historical land use influences the occurrence of some species based on their ecological preferences, these effects do not significantly influence community species richness and total composition.

**Supplementary Information:**

The online version contains supplementary material available at 10.1007/s10980-024-02016-6.

## Introduction

Effects of past land use and ecosystem management on present-day biodiversity of ecological communities have captivated ecologists for the past two decades (Foster et al. 2003; Perring et al. 2016; Garbarino and Weisberg 2020). There is growing evidence that both historical landscape structure and configuration (Lecoq et al. 2021; Scherreiks et al. 2022; Pan et al. 2022) and local historical land use (i.e., the type of land use characterizing the site or community of interest in the past; Bellemare et al. 2002; Gustavsson et al. 2007; Culbert et al. 2017) affect community composition and function. Consequently, historical management and land use have been put forth as an explanation of contemporary and future biodiversity patterns (Perring et al. 2016, 2018; Garbarino and Weisberg 2020; Boivin and Crowther 2021; Vilà-Cabrera et al. 2023).

There are various mechanisms behind historical land-use influences on present-day plant communities, and their importance depends on the duration of the historical management, the time span since its change, biodiversity facet (e.g., abundance, richness, composition) and the studied taxa (Foster et al. 2003; Flinn and Vellend 2005; Scherreiks et al. 2022; Pan et al. 2022). In plants, there are two pathways by which historical land use may leave imprints in current communities. First, historical habitats (or landscapes) might have harbored species that still occur under present-day conditions (i.e., biotic legacies), albeit under different management or land use (Levis et al. 1979; Verheyen et al. 2003; Svenning et al. 2008; Heubes et al. 2011; Karlík and Poschlod 2014). This is particularly relevant for certain plant species because they can exhibit lagged responses to landscape modifications facilitated by long-term in situ survival, persistent seed banks, and dispersal strategies favoring recolonization from neighborhood landscapes (Verheyen et al. 2004; Vellend et al. 2006). Consequently, the occurrence of certain plant species that are associated with specific conditions different from the current ones is often used as an indication of past habitat types or land uses, such as old-growth forests (so-called “ancient woodland indicators”; Wulf 1997; Verheyen et al. 2003) or ancient grasslands and cropland (Karlík and Poschlod 2014). Local introduction of crop or ornamental plants near historical human infrastructures are also important drivers of contemporary species occurrence patterns (Levis et al. 1979; Pärtel et al. 2007; Hejcman et al. 2013; Hlásná Čepková et al. 2016).

Second, there may be abiotic legacies of historic land use that shape current environmental conditions, influencing vegetation structure, ecosystem processes, and, ultimately, species diversity and composition (Cramer et al. 2008; Perring et al. 2016). Such legacies may include modification of the physical environment which result from various past anthropogenic activities (e.g., building construction, fertilization, plowing, soil drainage) and lead to diverging trajectories of change in contemporary abiotic conditions, such as soil structure and nutrient availability (Fraterrigo et al. 2005; Perring et al. 2016; Hájek et al. 2017; Mollier et al. 2022). Past abiotic factors also interact with biotic legacies, influencing both plant productivity (Glass et al. 2023) and regional species pools (Bruun et al. 2001; Cramer et al. 2008).

Consistent with these expectations, previous research suggests that historical land use explains a non-negligible portion of the variation in species richness and composition in contemporary forest vegetation (Svenning et al. 2008; Brudvig and Damschen 2011; Kelemen et al. 2014; Janssen et al. 2018; Shumi et al. 2018). However, for grasslands, these effects seem less obvious. While there is evidence that the maintenance of the connectivity and extent of grasslands on a landscape-scale over time positively supports plant diversity (Cousins 2009; Divíšek and Chytrý 2018; Scherreiks et al. 2022), the effects of local historical land-use type on community composition appear to be much weaker, as specifically shown in European grasslands (Pärtel et al. 1999, 2007; Bruun et al. 2001; Kuhn et al. 2021; but see Gustavsson et al. 2007; Hájek et al. 2017). However, limited availability of historical data often restricted research to small study areas and few historical land-use or grassland types (Heubes et al. 2011; Karlík and Poschlod 2014; Scherreiks et al. 2022).

Furthermore, while substantial research has focused on the effects of local historical land use on plant species diversity (such as species richness and beta diversity) (e.g., Bruun et al. 2001; Pärtel et al. 2007; Cousins 2009; Culbert et al. 2017; Janssen et al. 2018), little attention has been paid to studying how ecological and functional preferences of individual grassland species affect their response to historical land use (see e.g. Heubes et al. 2011), as previously observed in forests (Hermy et al. 1999; Kimberley et al. 2013; Kelemen et al. 2014). Analyses of this type can be based on species-level indicator values which bear information upon the realized niche optimum of an individual species along environmental (Ellenberg et al. 1992) and disturbance (Erdős et al. 2022; Midolo et al. 2023) gradients. Specifically, if land-use history affects current species occurrence, one should expect that contemporary vegetation harbors species that prefer conditions characteristic of historical land use. In addition, abiotic land-use legacies may still select for species based on their ecological preferences, e.g. when nutrient enrichment from previous agricultural sites favors more nutrient-demanding species in contemporary vegetation. At the same time, it is expected that pronounced changes in individual species occurrences will affect plant community composition, even though they may not directly alter species richness.

The recent digitization and interpretation of historical maps from the German (Krüger and Schnadt 2000; Walz 2002) and Austrian Empires (Timár et al. 2006; Skokanová et al. 2012) offer a unique opportunity to examine the effects of 19th-century land use on current vegetation. This, combined with the extensive contemporary vegetation data available in Central Europe (Chytrý and Rafajová 2003; Willner et al. 2012), allowed us to investigate the effects of 19th-century land use across an unprecedented geographic scope and range of habitat types. We leveraged this opportunity to test whether historical (i.e., 19th-century) land-use categories exert an effect on vascular plants of three broad habitat types (dry, mesic, and wet grasslands). We focused on three response metrics, namely: 1) species richness, 2) species composition, and 3) the occurrence of individual species in historical land-use categories and their ecological indicator values (i.e., the species’ niche optima describing their preference along environmental and disturbance gradients).

We asked the following questions: i) Do historical land-use categories explain current plant species richness and composition within each grassland habitat type? ii) Which plant species can be considered indicators of past land use, and are their ecological preferences consistent with the conditions characteristic of these land-use categories?

## Materials and methods

### Study area

**Fig. 1:**
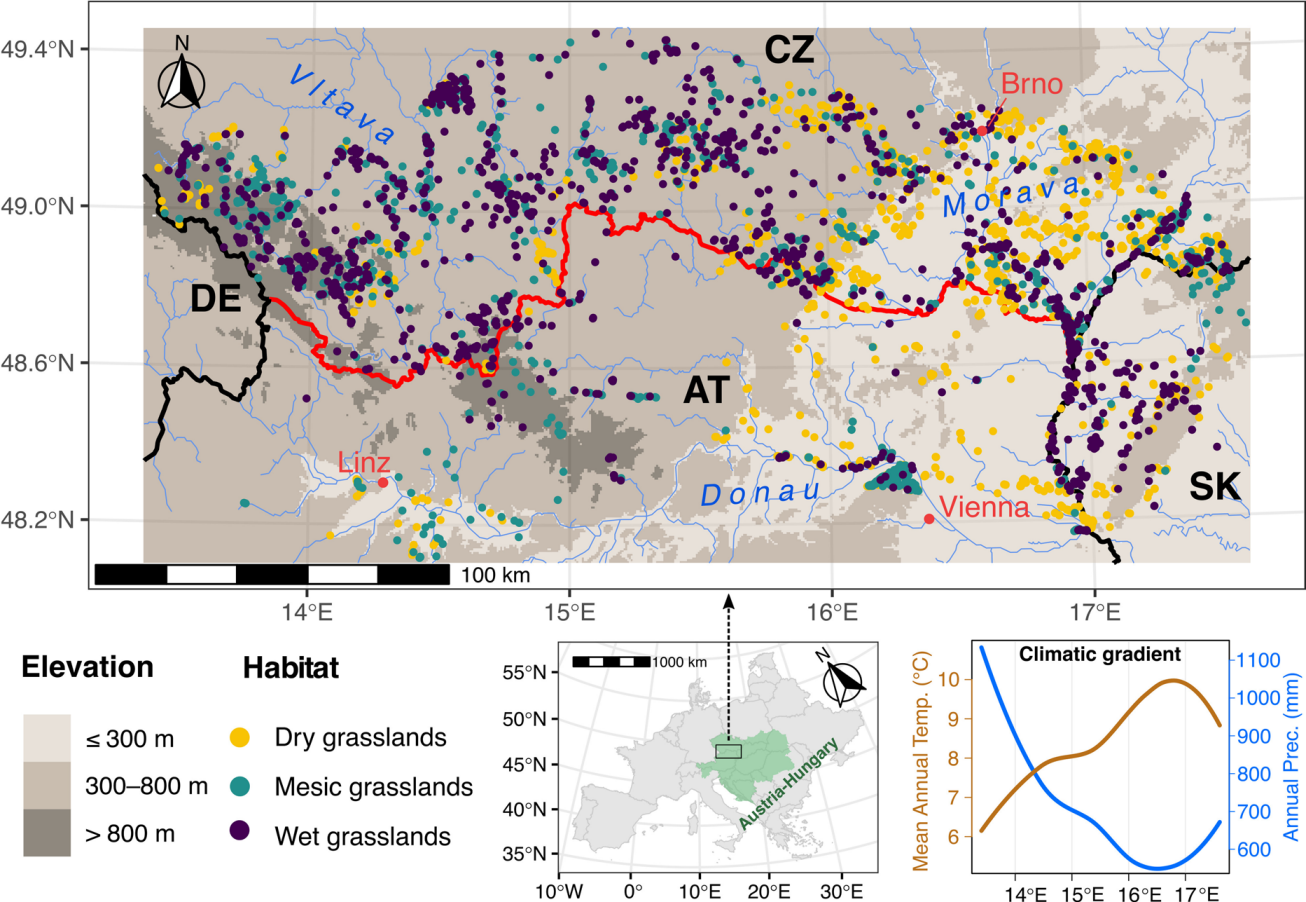
Study area showing the location of vegetation plots (colored points) used in the analysis. We selected plots in a 50-km buffer zone around the border between Austria and the Czech Republic (red line), as a part of the former Austro-Hungarian Empire. The map reports elevation, major rivers, drainage basins, and cities. The bottom-right panel displays the climatic gradient in terms of mean annual temperature and annual precipitation as functions of longitude at vegetation plot locations (predicted from loess regressions). Climatic data were retrieved from CHELSA (Karger et al. 2017a, b) at 1-km resolution

We defined the study area as a 50 km buffer along the border between Austria and the Czech Republic ranging from 13.3° E to 17.6° E longitude and from 48.1° N to 49.5° N latitude, including western Slovakia (as a part of former Czechoslovakia) but excluding southeastern Germany (Fig. 1). The study area spanned an elevational gradient from 100 to 1350 m, covering cold-to-warm and wet-to-dry longitudinal climatic gradients from west to east (Fig. 1).

The study area is a part of the former Empire of Austria (1804–1867) for which historical maps are available. This area offers a unique opportunity to study the effects of shared 19th-century land-use practices in Austria and Czechoslovakia, in which the landscape structure diverged significantly in the twentieth century. In particular, the collectivization of agricultural land in Czechoslovakia after 1948 led to distinct 20th-century land-use development changes. This combination of shared historical land use and contrasting recent landscape changes allowed us to test the long-term influence of historical land use in a context of varying economic and political history.

### Vegetation data

We based our analysis on vegetation plots from the European Vegetation Archive (EVA; Project 147; Chytrý et al. 2016; data retrieved in April 2022) sampled between 1946 and 2021. We focused on vascular plants classified at the level of species, subspecies, aggregates and hybrids (hereafter referred to as “species”). Bryophytes and lichens were excluded as they were not recorded in many plots. Taxa identified only to genus were treated as species solely for richness but excluded from composition and indicator species analyses. We unified species nomenclature following Euro + Med PlantBase (2024) and used species aggregates following the EUNIS-ESy system (Chytrý et al. 2020). The cover values (expressed in percentages) belonging to the same species following nomenclature corrections were summed within the same plot and vegetation layer. In those plots where the cover of the same species was recorded within different vegetation layers, we merged cover values following the formula proposed by Fischer (2015).

Our initial dataset included 12,869 grassland vegetation plots. However, to reduce the uncertainty of plot location, we excluded plots with unknown locations or uncertainty exceeding 500 m. Moreover, we only included plots with known sizes less than or equal to 100 m^2^. We did not include plots larger than 100 m^2^ to avoid sampling biases caused by potentially incomplete sampling of larger plots and because such plots were rare, creating potential outliers in the dataset for species richness and composition analyses. We classified the remaining plots into main habitat categories based on level 2 of the EUNIS classification system of European habitats (Chytrý et al. 2020) and discarded plots with no classification. Our final selection resulted in 6975 plots, classified into three main grassland habitat types:

1. Dry grasslands (EUNIS code “R1”; 3504 plots): species-rich grasslands with generally low productivity located on warmer and drier sites. In our study area, they were represented mostly by semi-natural and rocky calcareous grasslands located in the lowlands and the submontane areas. They were traditionally managed by grazing, and some semi-dry types also by hay-making.
2. Mesic grasslands (EUNIS code “R2”; 1603 plots): common grassland habitat representing meadows and pastures located on deep, well-drained soils in intermediate climatic conditions, mostly found at medium altitudes. In Europe, mesic grasslands are traditionally managed by grazing or for hay production because of their high productivity.
3. Wet (or seasonally wet) grasslands (EUNIS code “R3”; 1868 plots): including mesotrophic to eutrophic meadows and pastures of moist soils located in floodplains and brook valleys. Like mesic grasslands, they are often traditionally managed by grazing or for hay production.

We also included data from some of the forest fringe habitat category (“R5”) due to their similarity to the other grassland habitat types. Specifically, we assigned 491 plots classified as lowland moist or wet tall-herb and fern fringe (“R55”) to the wet grasslands category and 82 plots classified as thermophilous forest fringe of base-rich soils (“R51”) to the dry grassland category. Our final selection resulted in 1235, 5239, and 501 plots located within the modern borders of Austria, the Czech Republic, and Slovakia, respectively.

### Historical land-use data extraction

We used historical military maps from the former Austrian Empire, specifically Austrian military topographic maps from the “2nd military survey” at a scale 1:28 000, which were produced during the period 1819–1853. We retrieved maps georeferenced by Timár et al. (2006) via WMTS from the Arcanum web site (https://maps.arcanum.com). For the plots located in the Czech Republic (n = 5239), we extracted local historical land use from TopoLandUse data (Skokanová et al. 2012)—a database that includes vector land-use data for the whole Czech Republic from five periods based on different mapping sources. For the 1736 plots in Austria and Slovakia, we extracted historical land use by visual interpretation of the maps, employing the same methods and personnel as with the TopoLandUse data.

We distinguished six land-use categories: arable land, permanent grassland, forest, water body, settlement, and permanent crops. Arable land included all types of annual crops as well as fallow land and vegetable gardens. Permanent grassland (hereafter, grassland) included all types of grassland habitats (the historical map legend contained meadows, pastures, and wetlands) and sparsely vegetated areas (e.g., river sandbars and rock/gravel surfaces in mountain areas). Although different types of grassland habitats, such as meadows or wetlands, could be distinguished in the historical map, the quality of maps varied throughout the study area, making it difficult to capture the types precisely. Therefore, all grassland types were grouped into one category. Forests included all types of forests and scrub vegetation, as the historical map legend did not distinguish among different woody vegetation types. Water bodies consisted of both man-made and natural features (lakes, ponds, and pools were distinguished in the historical map legend). Settlements consisted of buildings and adjacent gardens, both distinguished in the historical map legend. Finally, permanent crops included fruit orchards, vineyards and, in some cases, also hop-fields (all distinguished in the historical map legend).

After we had assigned each plot to one of the six historical land-use categories, we excluded combinations of categories and grassland habitat types (dry, mesic, wet) with less than 20 plots per historical land-use category and grassland habitat type to avoid highly unbalanced data. Thus, we did not consider the “water body” historical category for dry and mesic grasslands and “permanent crop” historical category for wet grasslands. Across the final selection of 6975 plots, the following numbers of plots were classified in these historical land-use categories: 1462 as “arable land”, 1093 as “forest”, 3592 as “grassland”, 462 as “permanent crop”, 264 as “settlement”, and 102 as “water body”. Each of the three current grassland habitat types had different counts for each historical land-use category (Figure S1.1; Online Appendix S1).

Finally, to quantify the change in land use over time, we compared the historical land-use category at each plot location to the corresponding land cover type for the year 2018, extracted from the CORINE Land Cover (CLC) + Backbone (Copernicus Land Monitoring Service 2024). This analysis allowed us to assess the distribution of plots that potentially underwent land cover changes or have similar land use between today and the past in Austria and the former borders of Czechoslovakia (see Figure S1.3; Online Appendix S1).

### Environmental predictors

Because plant diversity and composition are influenced by contemporary climate and soil conditions, which also likely played a role in shaping historical natural habitats and anthropogenic land use in the study area (Skokanová et al. 2016), we standardized for variation of these conditions by incorporating them as predictors of species richness and composition. Contemporary environmental predictors were obtained from 19 bioclimatic variables at 1 km resolution from CHELSA (Karger et al. 2017a, b) and soil pH in H_2_O (estimated at 5–15 cm depth from the soil surface) at 250 m resolution from SoilGrid250m (Hengl et al. 2017). To address high collinearity, we discarded variables with an absolute Pearson correlation coefficient ≥ 0.7 using a stepwise procedure that removed variables with the highest Variance Inflation Factor (VIF) at each step, with the “vifcor” function of the *usdm* R package (Naimi et al. 2014). The variables selected for analysis were mean diurnal air temperature range (“bio2”), isothermality (“bio3”), mean daily mean air temperatures of the wettest quarter (“bio8”), mean daily mean air temperatures of the driest quarter (“bio9”), precipitation seasonality (“bio15”), and soil pH. In addition, we controlled for species-area relationship by including plot size as a log-transformed covariate in the models of species richness. Finally, to account for historical and socioeconomic differences between Austria and former Czechoslovakia in the second half of the twentieth century, we included these two countries as a categorical predictor of species richness and composition.

### Analysis of species richness

We used both generalized linear mixed models (GLMM) and Random Forests to relate species richness to historical land-use and environmental covariates within each habitat type separately (question I). To control for overdispersion in the GLMMs, we utilized observation-level random effects (OLRE) (Bulmer 1974; Harrison 2014). The model adjusts for overdispersion by capturing individual-level variability in the data, allowing for a more accurate estimation of variance and accommodating deviations beyond what is expected under generalized linear models. We assumed a Poisson distribution of the response variable (using a log-link function) implemented via the “glmer” function in the *lme4* R package (Bates et al. 2015). We included quadratic terms for all continuous predictors in the GLMMs to account for potential nonlinear responses. Random Forests were fitted using the “randomForest” function from the *randomForest* R package (Liaw and Wiener 2002). To build the Random Forest models, we fitted 500 trees with 3 randomly sampled variables as candidates at each split. We trained the models with a set of 2/3 of the data and used a randomly chosen one-third of the available explanatory variables. The remaining one-third of the data was kept out-of-bag as a testing set and used to evaluate the model’s predictive performance. Partial dependence of each predictor in GLMM and Random Forest was obtained using the “partial” function of the *pdp* R package (Greenwell 2017) to depict the marginal effect of a given predictor on the mean changes in species richness (see also Figure S2.1; Online Appendix S2).

### Analysis of species composition

We used canonical correspondence analysis (CCA; ter Braak 1986; Legendre and Legendre 2012) to assess the land-use history effects on species composition in each habitat type (question I). We conducted CCA using the “cca” function of the *vegan* R package (Oksanen et al. 2022). We first generated a distance matrix using Bray–Curtis dissimilarity calculated on species relative cover ranging between 0 and 1. After calculating the Bray–Curtis dissimilarity, we Hellinger-transformed the distance matrix before performing CCA (Legendre and Gallagher 2001) using the “decostand” function of the *vegan* R package. In a separate analysis, we also evaluated the importance of each individual predictor in terms of variation explained (R^2^) by fitting all predictors together in a PERMANOVA using the “adonis2” function of the *vegan* R package with 100 permutations (Table S2.1; Online Appendix S2).

### Variation partitioning

To distinguish between the effects of historical land use and environmental predictors on species composition and richness, we conducted variation partitioning (Borcard et al. 1992; Viana et al. 2022). We compared R^2^ values from the GLMMs, Random Forest (for species richness), and CCA (for species composition) including 1) historical land use and environmental predictors together, 2) historical land use only, and 3) environmental predictors only. Environmental predictors included climatic variables, soil pH, and country (accounting for differences in management between the two countries). For species richness, plot size was always included in each model as a key covariate. We then used these models to partition the total explained variance into variation explained independently by the two sets of predictors and the combination of both, following Viana et al. (2022). For Random Forest, we used the pseudo-R^2^ values of the model trained on the entire dataset calculated as 1—[*MSE*/var(*S*)], where *MSE* represents the mean squared error and var(*S*) denotes the variance of the observed values of the response variable (species richness, *S*). For GLMMs, we used the coefficient of determination for generalized linear models proposed by Zhang (2017) estimating the proportion of variation explained by the fixed-effects factors using the “rsq.glmm” function from the *rsq* R package (Zhang 2023). For CCA, we adjusted R^2^ values according to the number of predictors (Peres-Neto et al. 2006) using the “RsquareAdj” function from the *vegan* R package.

### Ecological and disturbance indicator values

We used species-level indicator values as proxies estimating the ecological preferences (realized niche optima) of individual species along five environmental gradients (i.e., light, temperature, soil moisture, soil nutrients, and soil reaction) (Ellenberg et al. 1992) and five disturbance gradients (i.e., disturbance frequency, disturbance severity, grazing pressure, mowing frequency, and soil disturbance) (Midolo et al. 2023). Ellenberg-type indicators summarize realized niche optima of each species via regional expert-based indicator value systems. We retrieved Ellenberg-type indicator values from Tichý et al. (2023), which reported the original country-level indicator value databases for Austria (Karrer 1992) and the Czech Republic (Chytrý et al. 2018). We used country-level indicator values to better match specific local conditions of the study area. For species with different indicator values for Austria and the Czech Republic, the values were averaged. Disturbance indicator values were retrieved from Midolo et al. (2023) and reflect the disturbance levels of European habitats in which a given species frequently occurs. For the subsequent analyses, we only selected species for which both environmental and disturbance indicator values were given in the specified sources. This selection resulted in a total of 1461 species out of the initial 1573 species in our dataset. The 112 discarded species were infrequent in the vegetation data.

Since ecological indicator values were coordinated, we conducted a principal component analysis (PCA) on the nine indicator variables with scaled and centered data. Subsequently, we restricted our analysis of indicator values to the first four orthogonal axes, which explained more than 80% of the total cumulative variation of indicator values (following “varimax” rotation; Kaiser 1958) (see Online Appendix S3).

### Indicator species analysis

We conducted a species-level analysis to test whether species associated with specific historical land-use categories exhibit ecological niche optima consistent with those categories (question II). We first applied an indicator species analysis, measuring the group-size corrected indicator value index (“IndVal.g”; hereinafter referred to as IndVal) [Dufrêne and Legendre (1997); extended by De Cáceres et al. (2010)]. For this purpose, we used the “multipatt” function of the *indicspecies* R package (De Cáceres and Legendre 2009) using historical land-use category of each plot as the site grouping variable. The “multipatt” function computes an IndVal index for each species in each group, i.e. historical land-use category, assigning each species to the category with the highest IndVal. The IndVal statistic ranges from 0 to 1, representing the degree of association (from low to high, respectively) of the species with each category. IndVal equals 1 when all records of a species are found in a single category and when the species occurs on all sites of that category (De Cáceres et al. 2010). The significance of IndVal was determined using 999 random permutations. We calculated IndVal values separately for each habitat type. Additionally, to identify unique taxa for each combination of both habitat type and historical land use, we conducted a second calculation for each of these combinations across the entire dataset (see Table S3.1; Online Appendix S3).

For each habitat, we tested for differences in ecological and disturbance indicator values between groups of species associated with the different historical land-use categories. We conducted these analyses separately for each of the selected principal component axes. Namely, we used the values of the species on these axes as the response variable and the historical land-use categories as predictors in a one-way ANOVA. We thereby weighed each species’ score by the IndVal statistic to assign greater weight to the species with the strongest association with a specific historical land-use category and downweighted those species whose association with a given historical land-use category was low. We also investigated whether species significantly associated with a given historical land use are characteristic of current EUNIS habitat types other than grasslands that are common in the study area (see Table S1.1; Online Appendix S1), namely forest, scrub, vegetated man-made, and wetland habitats. To do so, we used constant, diagnostic, and dominant species lists from the EUNIS Habitat Classification system and habitat distribution maps (Chytrý et al. 2020) available at the FloraVeg.EU website (https://floraveg.eu/).

## Results

### Species richness

**Fig. 2:**
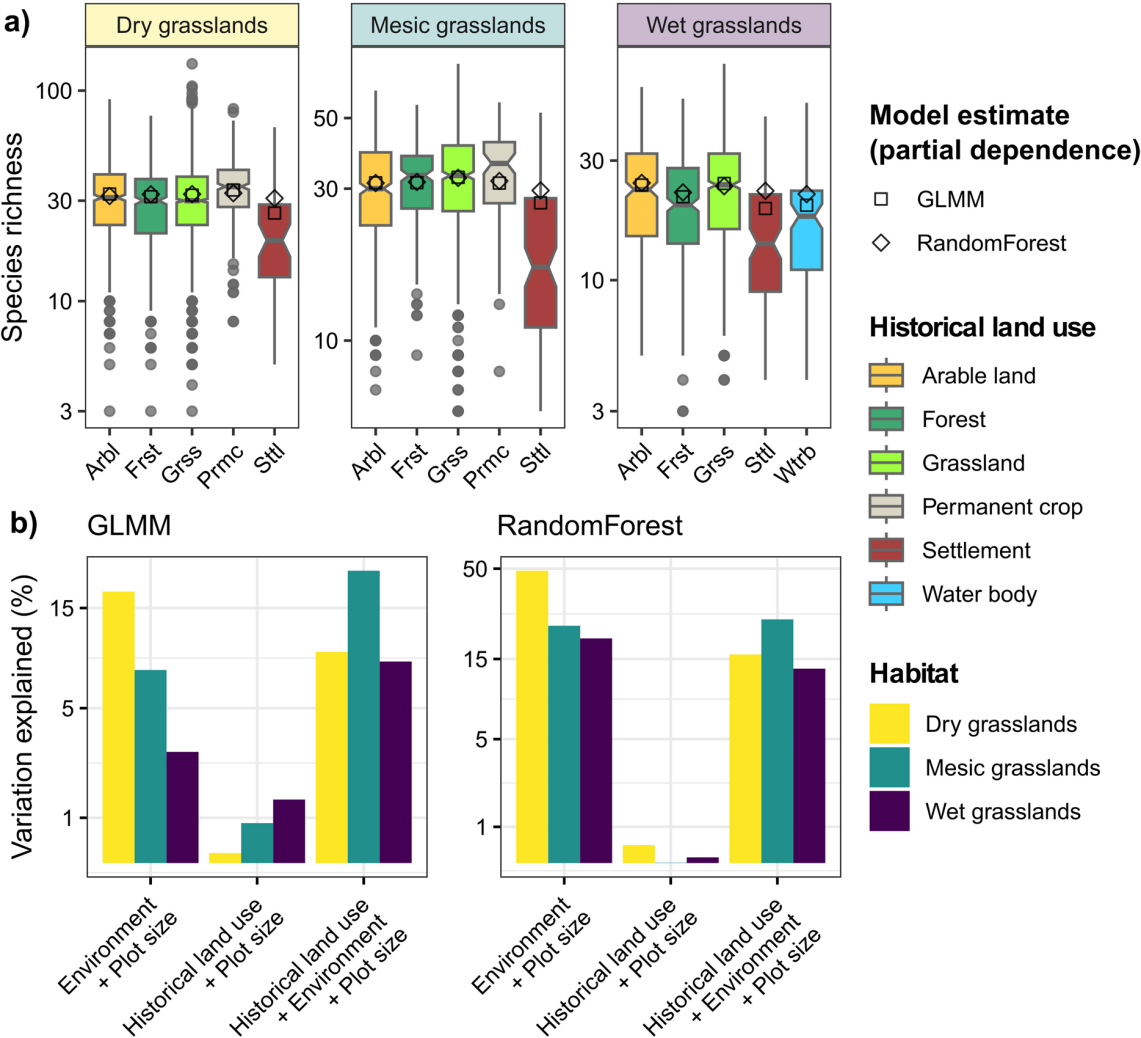
Species richness across grassland plots, categorized by historical land use (panel **a**). Boxplots summarize the distribution of species richness within each category. The boxes represent the central 50% of the data, with the line inside depicting the median and the notches indicating the confidence interval of the median. Additionally, the panel includes partial dependence estimates of historical land-use effects on species richness obtained from generalized linear mixed models (GLMM) and Random Forest models, where all other predictors are held at their mean. Panel **b)** depicts the variation (explained variance, R^2^) partitioned into the contributions of each component in the GLMM and Random Forest models. Variation partitioning is obtained by comparing the R^2^ values of models’ fit with historical land-use categories only, environmental predictors only, and a combined model that includes both sets of variables

We found no significant influence of local historical land use (as documented in the military maps spanning from 1819 to 1853) on species richness across the three grassland habitats studied. Species richness models (GLMMs and Random Forests) highlighted negligible differences across historical land-use categories (Fig. 2a), and variation partitioning revealed a consistently higher contribution of plot size and some environmental predictors (Fig. 2b; see also Random Forest variable importance in Figure S2.3; Online Appendix S2). Specifically, in all habitat types, richness peaked at medium intervals of precipitation seasonality (“bio15”) (Figure S2.1-S2.2; Online Appendix S2). Nonetheless, lower species richness was observed in plots formerly located in settlements than in other categories, but this effect was not significant once other covariates were included in the model (Fig. 2a).

### Species composition

**Fig. 3:**
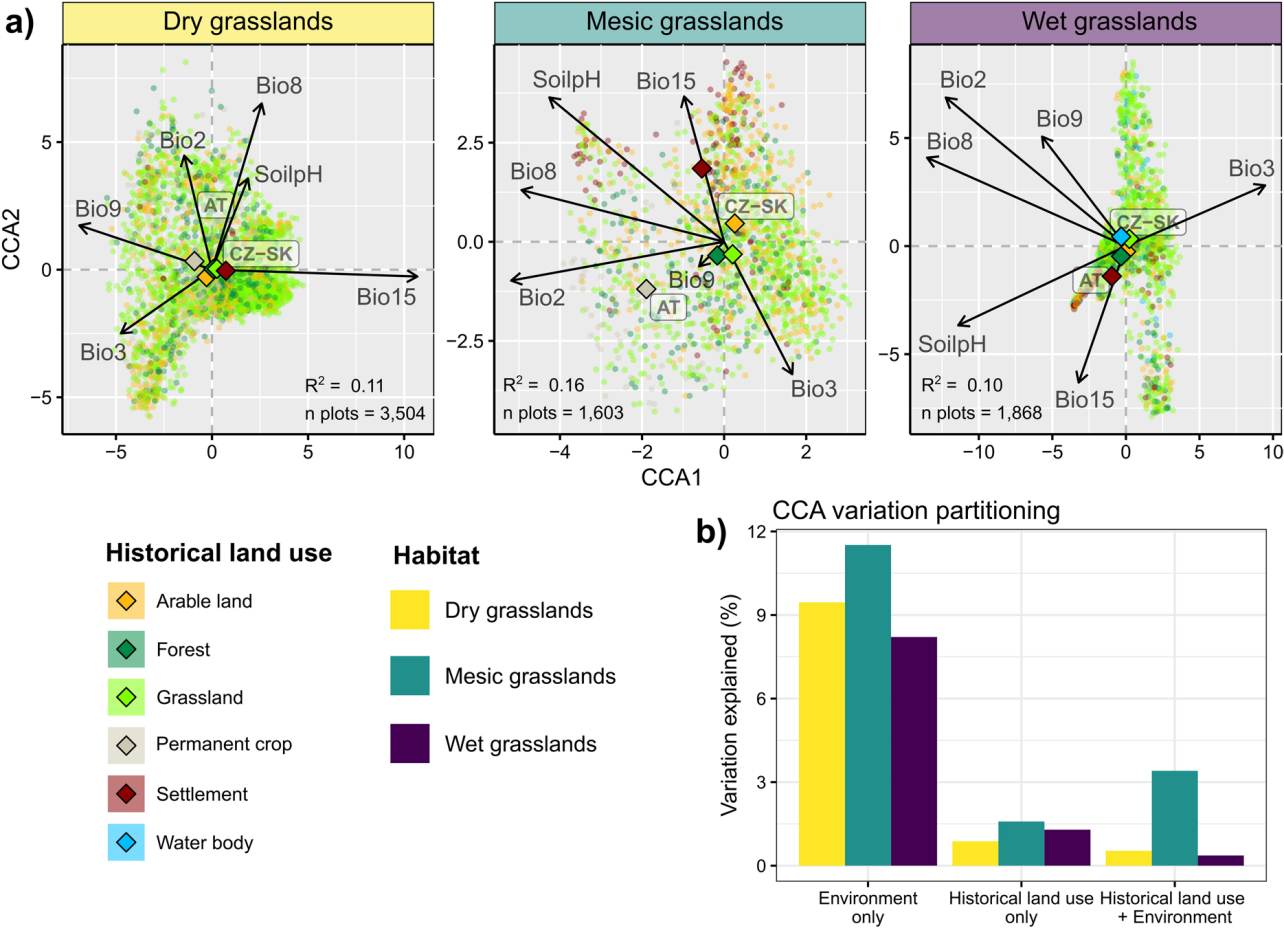
Biplot of the correspondence analysis (CCA) including both historical land-use categories and environmental predictors (= panel **a**) and results of the variation partitioning between historical land-use categories and environmental predictors (= panel **b**) for each habitat category (= level 2 EUNIS habitat). Smaller dots displayed in panel **a**) correspond to individual vegetation plots. Variation explained by each component (panel **b**) was calculated by comparing adjusted R^2^ values from CCA fitted with historical land-use categories and environmental predictors separately, and a combination of both. Environmental predictors are mean diurnal air temperature range (“bio2”), isothermality (“bio3”), mean daily mean air temperatures of the wettest quarter (“bio8”), mean daily mean air temperatures of the driest quarter (“bio9”), precipitation seasonality (“bio15”), and soil pH, soil pH, and country (“AT”, Austria vs. “CZ-SK”, the Czech Republic and Slovakia)

Ordination analysis (CCA, Fig. 3a) highlighted a moderate differentiation in species composition among the most common historical land-use categories. The only exception was a distinct species composition of current mesic grasslands occurring on sites of former permanent crops and settlements (Fig. 3a). Similarly to species richness, we found only a small fraction of the variation in species composition uniquely explained by historical land-use categories across all three habitat types (< 1.5%) (Fig. 3b). Mesic grasslands showed a slightly higher portion of variation explained jointly by historical land use and environmental variables, potentially because of the coupling between historical settlement location and climate (i.e., “bio15”; precipitation seasonality) (Fig. 3a). Nonetheless, variation partitioning showed that environmental variables alone explained much larger, albeit still relatively low (11.5%), portions of variation than historical land use. The PERMANOVA analyzing the effects of individual predictors (Table S2.1; Online Appendix S2) showed that the highest relative importance (sum of squares) was found for the mean diurnal range of temperatures (“bio2”) in all habitat types except for dry grasslands, where precipitation seasonality (“bio15”) had greater importance.

### Indicator species

Considering each grassland habitat separately, we identified a total of 492 out of 1,498 species significantly associated with historical land-use categories (IndVal statistics ranging from 0.06 to 0.59; p-value < 0.05). Upon comparing linkages to historical land use with current associations to common habitats found in the study area (Table 1), we found several species whose current characteristic habitats did not match the historical land use to which they were assigned. For example, species typical of grassland habitats, such as *Alopecurus pratensis* and *Centaurea scabiosa* were assigned to historical arable land and permanent crops, respectively. However, certain species consistently exhibited a match between the historical land use and their current characteristic habitats (Table 1). For instance, some species that occur both in grasslands and forests were associated with historically forested sites (e.g., *Impatiens noli-tangere* and *Teucrium chamaedrys*). Similarly, grassland species that are also frequent in some man-made vegetated habitats were associated with historical arable land (e.g., *Poa pratensis* aggr., *Plantago lanceolata*), settlements (e.g., *Arenaria serpyllifolia*, *Lolium perenne*, *Taraxacum* sect. *Taraxacum*, *Plantago major*) and permanent crops (*Arrhenatherum elatius*).

**Table 1:**
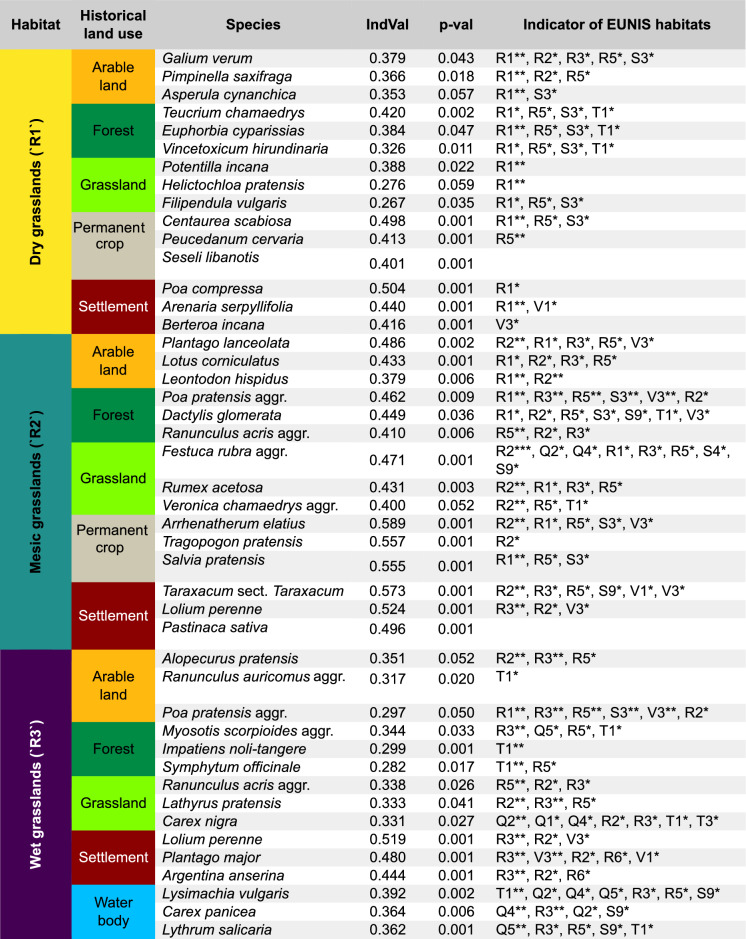
List of the three species with the highest and significant (p-val < 0.05) indicator value (“IndVal”) for each historical land use, estimated separately within each habitat type (“Habitat”)

The table includes the level-2 EUNIS habitats indicating whether a species is *constant* (*), *diagnostic* (**), or *dominant* (***) for at least one level-3 habitat within each level-2 class. Only level-3 habitats common in the study area were considered (see Table S1.1) and grouped under level 2, encompassing grasslands (“R”), scrub (“S”), forests (“T”), wetlands (“Q”), and vegetated man-made habitats (“V”). See.csv files in the Supplementary Material for complete IndVal statistics of all the species analyzed

Species type: * = Constant species; ** = Diagnostic species; *** = Dominant species. N.B.: If a species is classified as both 'diagnostic' and 'constant', only 'diagnostic' is indicated. Similarly, if a species is classified as both 'dominant' and 'constant,’ only 'dominant' is indicated

Level-2 EUNIS habitats: Q1 = “Raised and blanket bogs”; Q2 = “Valley mires, poor fens and transition mires”; Q4 = “Base-rich fens and calcareous spring mires”; Q5 = “Helophyte beds”; R1 = “Dry grasslands”; R2 = “Mesic grasslands”; R3 = “Seasonally wet and wet grasslands”; R5 = “Woodland fringes and clearings and tall forb stands”; R6 = “Inland salt steppes and salt marshes”; S3 = “Temperate and Mediterranean-montane scrub”; S4 = “Temperate heathland”; S9 = “Riverine and fen scrub”; T1 = “Broadleaved deciduous forests”; T3 = “Coniferous forests”; V1 = “Arable land and market gardens”; V3 = “Artificial grasslands and herb-dominated habitats”

**Fig. 4:**
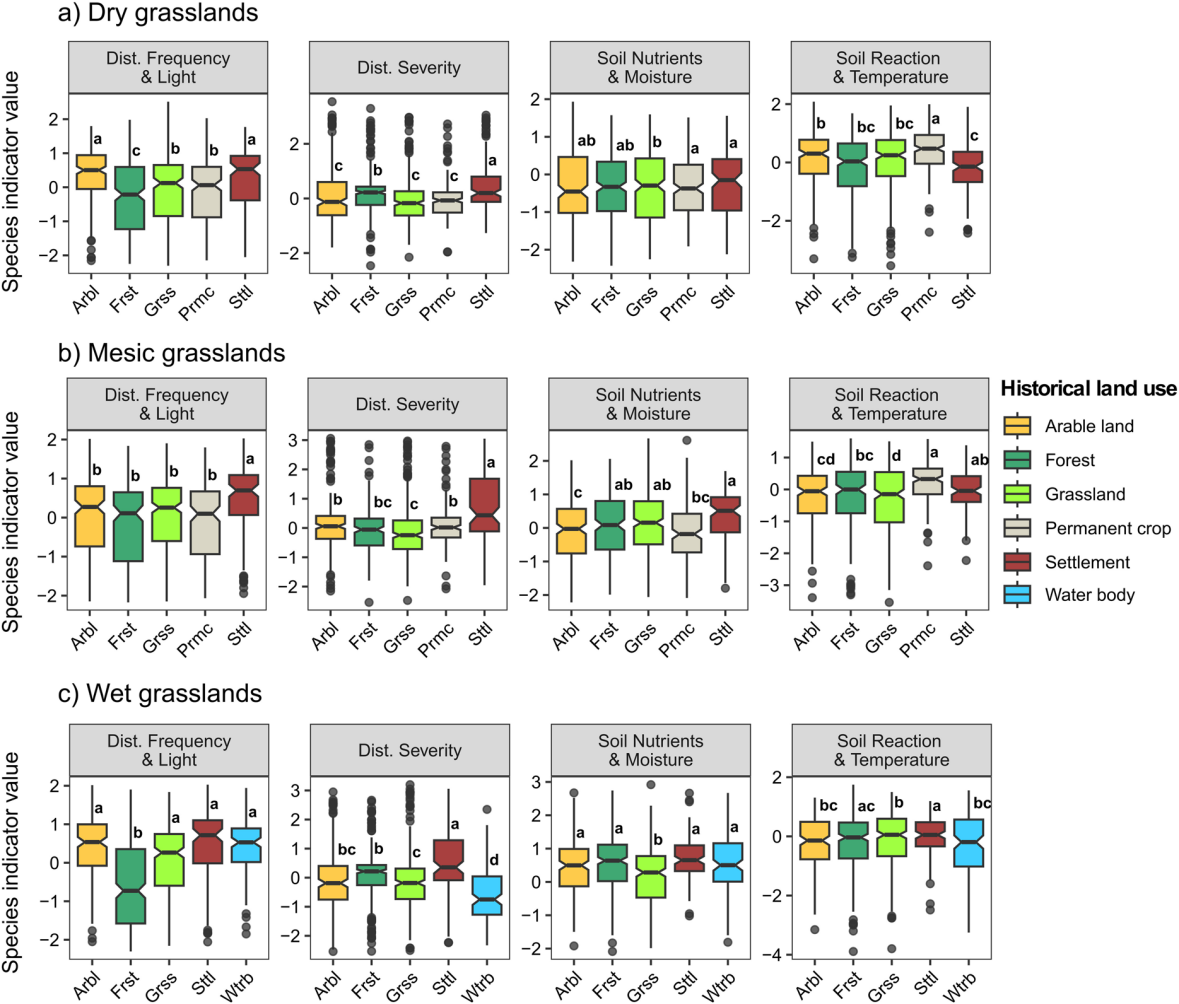
The distribution of ecological preferences (PCA axes; y-axis) of species assigned to different historical land-use categories based on indicator species analysis (IndVal) for the three grassland habitat types analyzed (dry, mesic, and wet grasslands). The first four axes are shown in each panel, corresponding to 1) disturbance frequency and light, 2) disturbance severity, 3) soil nutrients and soil moisture, and 4) soil reaction and temperature. Letters on each box depict the results from Tukey's test, estimating differences across groups obtained from IndVal-weighted ANOVA. Each box represents the central 50% of the data, with the line inside depicting the median and the notches indicating the confidence interval of the median

Consistently, the comparisons of species-level ecological indicator values with associations to historical land-use categories reflected some environmental and disturbance conditions of these historical land-use categories (Fig. 4). Specifically, species more frequently found on historically forested sites exhibited significantly lower indicator values of disturbance frequency, mowing frequency, and light availability compared to species in other categories, except for the species in mesic grasslands (Fig. 4b). Across all habitat types, species associated with historical settlements were characterized by significantly higher disturbance severity indicator values, and wet grasslands located on historical water bodies displayed significantly lower disturbance severity indicator values.

## Discussion

### Historical effects on plant species richness and composition: causes and limitations

Our results on community diversity and composition were not fully consistent with findings from previous research reporting substantial predictive power of local historical land use on the plant diversity of European vegetation (e.g., Bruun et al. 2001; Cousins and Eriksson 2002; Gustavsson et al. 2007; Svenning et al. 2008; Kuhn et al. 2021). Several factors may explain this discrepancy. First, the most striking historical effects are usually reported in forests (e.g., Flinn and Vellend 2005; Svenning et al. 2008; Brudvig and Damschen 2011). Forests represent a late successional stage where legacies from previous land uses (like arable land or grassland) can influence species composition in the understory for many decades due to less frequent disturbance. Additionally, forest herbs, which are the main contributors to temperate forest richness, are particularly sensitive to land-use change and fragmentation because of their low colonization and dispersal ability (Hermy et al. 1999; Flinn and Vellend 2005). Both factors contribute to the fact that historical land-use and management legacies remain detectable in forests for a long time (Perring et al. 2016). Grasslands, conversely, display faster species turnover and colonization rates (see e.g., Sojneková and Chytrý 2015) following land-use conversion or management intensification, driven by more selective disturbance regimes like mowing and grazing. These strong impacts potentially cancel out the effects of past legacies more quickly. In line with our results, Němec et al. (2022) found little impact of 70-year-old historical land use on plant diversity in grasslands of the southern Czech Republic, while they detected more noticeable effects from more recent (20 years) land use. This is consistent, for example, with historical trends of some mesic and moist grasslands experiencing silage production intensification starting from the 1950s in Western (Boch et al. 2020) and Eastern (Török et al. 2020) European countries. Indeed, land-use intensification and eutrophication during the second half of the twentieth century have selected a limited pool of competitive and nutrient-demanding species in European floras (Wesche et al. 2012; Klinkovská et al. 2024).

The spatial extent of our study area is also a factor that may reduce the effects of centuries-old historical land use on vegetation. To our knowledge, our study encompasses a much broader environmental gradient compared to previous studies assessing historical land-use effects on vegetation. However, across a large geographic extent, land use is partially a result of climate and topography (Thuiller et al. 2004), which also influence land-use change dynamics (Skokanová et al. 2016). Therefore, gradients over large geographic extents are presumably more likely to capture part of the explained variation in species richness and composition that may be attributable to land use. This is supported by our variation partitioning results, which showed an intermediate amount of variation explained jointly by both historical land use and environmental predictors (Fig. 2b). This highlights the importance of geographic extent when considering the effects of land use on vegetation, with historical land use becoming better detectable at smaller spatial extents and under reduced environmental heterogeneity.

The spatial resolution at which we looked at historical land use likely played a role as well. Indeed, studies attributing significant variation in species diversity to historical drivers in grasslands have focused on landscape-level metrics (e.g., historical grassland connectivity) as key predictors of species richness (e.g., Cousins 2009; Scherreiks et al. 2022), rather than local historical land-use type at plot location. In theory, the resolution at which landscape characteristics affect biological responses within a local plot (referred to as the landscape “scale of effect”; Jackson and Fahrig 2012) is expected to increase as we move from individual occurrences to community diversity (Miguet et al. 2016). Our results are consistent with this expectation, as the plot-level historical land use assessed here influenced the occurrence of some species but not of species diversity metrics. Additionally, landscape at fine resolution is expected to influence habitat specialists with lower colonization ability, while landscape configuration at larger buffer sizes is expected to better capture processes related to generalist, long-distance dispersed species with higher colonization ability (Miguet et al. 2016; Scherreiks et al. 2022). Because grassland habitats may harbor more species dispersed by wind over long distances than closed-canopy habitats (Lorts et al. 2008), or, in general, with a higher colonization ability in general (Sojneková and Chytrý 2015), it is possible that landscape-level metrics measured on large buffer sizes, rather than local land-use categories, may better contribute to explaining plant community patterns in grassland habitats. Furthermore, besides capturing key ecological processes, landscape-level metrics calculated over larger neighborhoods have the implicit advantage of reducing potential bias of geographic coordinate uncertainty affecting both old historical maps and vegetation plots. However, the lack of fully vectorized digital maps in Austria and Slovakia prevented us from incorporating these landscape-level metrics in our analysis.

Our results were limited by the static nature of the land-use data, as we lacked information on land-use trajectories between multiple time steps (see e.g., Gustavsson et al. 2007), potentially missing transient changes that could influence plant diversity in the vegetation. Furthermore, although we detected no striking differences between Austria and the Czech Republic, our study took place in an area with contrasting and relatively recent historical socio-economic changes influencing land use (Němec et al. 2022), unlike grassland studies conducted in regions with longer political and land-use stability, such as Sweden (Cousins and Eriksson 2002; Gustavsson et al. 2007) and Western Germany (Scherreiks et al. 2022). For example, some Czech and Slovak grasslands have likely been converted to and from arable land more than once over the course of the twentieth century, following changes before and after agricultural collectivization, potentially masking the true impact of older land use (Sychrová et al. 2024).

### Signals of historical legacies from individual species

The indicator value analysis provided support for the hypothesis of historical legacies in our data. This evidence comes from the association between some historical land uses and species-specific occurrence patterns in contemporary vegetation, as well as the ecological properties of these species. For example, *Arrhenatherum elatius* was more often associated with grasslands located on sites historically used for planting permanent crops. This result partially reflects the ecology of this species as described by European botanists of the seventeenth-eighteenth century, identifying *A. elatius* as a widespread plant of vineyards and orchards before it became the most common grass of European mesic grasslands (Poschlod et al. 2009). Historically, nutrient-poor European grasslands offered a less suitable habitat for *A. elatius* compared to occasionally fertilized permanent crops. As overall nutrient levels in grasslands have increased, these areas have become more suitable for *A. elatius*, contributing to its current widespread dominance. Similarly, the association between *Plantago lanceolata* and former arable land generally reflects the connection between this plant and agricultural land use in Europe starting from the Neolithic (Deza-Araujo et al. 2022). While *P. lanceolata* is more frequently found in grasslands today than in the past, it is possible that it was historically more common on arable land during the nineteenth century, when less effective ploughing techniques were used. In general, species significantly associated with specific historical land uses may have persisted locally due to remnants of past conditions or an extinction debt. However, they may also be assigned to certain historical land uses for other reasons, such as current conditions fulfilling their requirements. Therefore, we caution against the uncritical use of these species to retrospectively identify past land use in present-day vegetation, especially outside our study area.

Although we found that the occurrence of more than 30% of the species present in the vegetation surveyed in our study area was related to historical land use, the lack of a clear effect on species composition and richness seems to suggest that the historical influence does not encompass a sufficiently large number of species to scale up at the community level. Nonetheless, our results comparing ecological indicator values among individual species highlighted the potential for tracking some historical legacies that still influence species occurrence through species’ disturbance preferences, specifically from historic forests, settlements, and water bodies. On the other hand, the lack of a clear difference in the ecological and disturbance indicator values of species assigned to historical grasslands, arable land, and permanent crops may reflect the recurring conversions between grasslands and croplands that characterize recent land-use dynamics in Europe (Fuchs et al. 2015; Pazúr et al. 2024), as well as the rapid colonization of abandoned arable land by grassland species from neighboring landscapes (Sojneková and Chytrý 2015). This makes it difficult to disentangle potential biological impacts without more detailed comparisons of temporal series. In addition to indicator values, further relevant species attributes not assessed here are dispersal ability and life history traits influencing local persistence strategies, which could explain the influence of landscape and management changes on plant communities (Damschen et al. 2014; Scherreiks et al. 2022; Martello et al. 2023). To this end, a previous study employing indicator analysis on forest vegetation in Central Europe (Kelemen et al. 2014) identified functionally distinct groups based on individual species occurrences on sites with different management histories.

## Conclusions

We investigated the potential influence of historical land use 100 to 200 years ago on Central European grassland vegetation. Although we found such an effect, it was weaker than reported in other studies. Our results suggest a hierarchical influence, with nineteenth-century legacies being most detectable via the persistence of species indicative of historical land uses, followed by total species composition, while leaving almost no trace in species richness. This indicates that signatures of specific historical land-use conversions, including the restoration of grasslands in land previously used for other purposes, may still be detectable even after two centuries. However, while 19th-century land use can leave a legacy via the occurrence of some species, community metrics such as diversity and composition are less affected in the long term. Understanding these dynamics is crucial not only for conservation actions but also for broader management practices, depending on whether these target individual species or emerging properties of plant communities. In addition, potential historical legacies revealed by our indicator analysis may influence other equally relevant ecosystem processes, such as the diversity of other taxa and associated ecosystem services.

## Supplementary Information

Below is the link to the electronic supplementary material.

Supplementary file1 (DOCX 2018 KB)

## Data Availability

Full data and R code are accessible at the Figshare data repository: 10.6084/m9.figshare.26305393.v2.
