# Supplementary material for: Nineteenth-century land use shapes the current occurrence of some plant species, but weakly affects the richness and total composition of Central European grasslands
Source: Landsc Ecol. 2025 Jan 13;40(1):22. doi: 10.1007/s10980-024-02016-6 (PMC11729212; doi:10.1007/s10980-024-02016-6)
Supplement: Supplementary file 1 — Supplementary file1 (DOCX 2018 KB) [file 10980_2024_2016_MOESM1_ESM.docx]

# Supplementary Information

**Journal**: *Landscape Ecology***Title**: "*Nineteenth-century land use shape the current occurrence of some plant species, but weakly affects richness and total composition of Central European grasslands*"
**Authors**: Midolo, Gabriele^1*^; Skokanová; Hana^2^; Clark, Adam Thomas^3^; Vymazalová, Marie^2^; Chytrý, Milan^4^; Dullinger, Stefan^5^; Essl, Franz^6^; Šibík, Jozef^7^; Keil, Petr^1^

**Affiliations**: **1** = Department of Spatial Sciences, Faculty of Environmental Sciences, Czech University of Life Sciences Prague, Praha-Suchdol, Czech Republic; **2** = Silva Tarouca Research Institute for Landscape and Ornamental Gardening, Department of Landscape Ecology, Brno, Czech Republic; **3** = Department of Biology, University of Graz, Graz, Austria; **4** = Department of Botany and Zoology, Faculty of Science, Masaryk University, Brno, Czech Republic; **5** = Division of Biodiversity Dynamics and Conservation, Department of Botany, University Vienna, Vienna, Austria; **6** = Division of BioInvasions, Global Change & Macroecology, Department of Botany and Biodiversity Research, University of Vienna, Vienna, Austria; **7** = Plant Science and Biodiversity Center, Slovak Academy of Sciences, Bratislava, Slovakia
*** Correspondence** Gabriele Midolo; e-mail: [midolo@fzp.czu.cz](mailto:midolo@fzp.czu.cz); ORCID: <https://orcid.org/0000-0003-1316-2546>

# **Appendix S1: Habitat type and historical land use information**

**
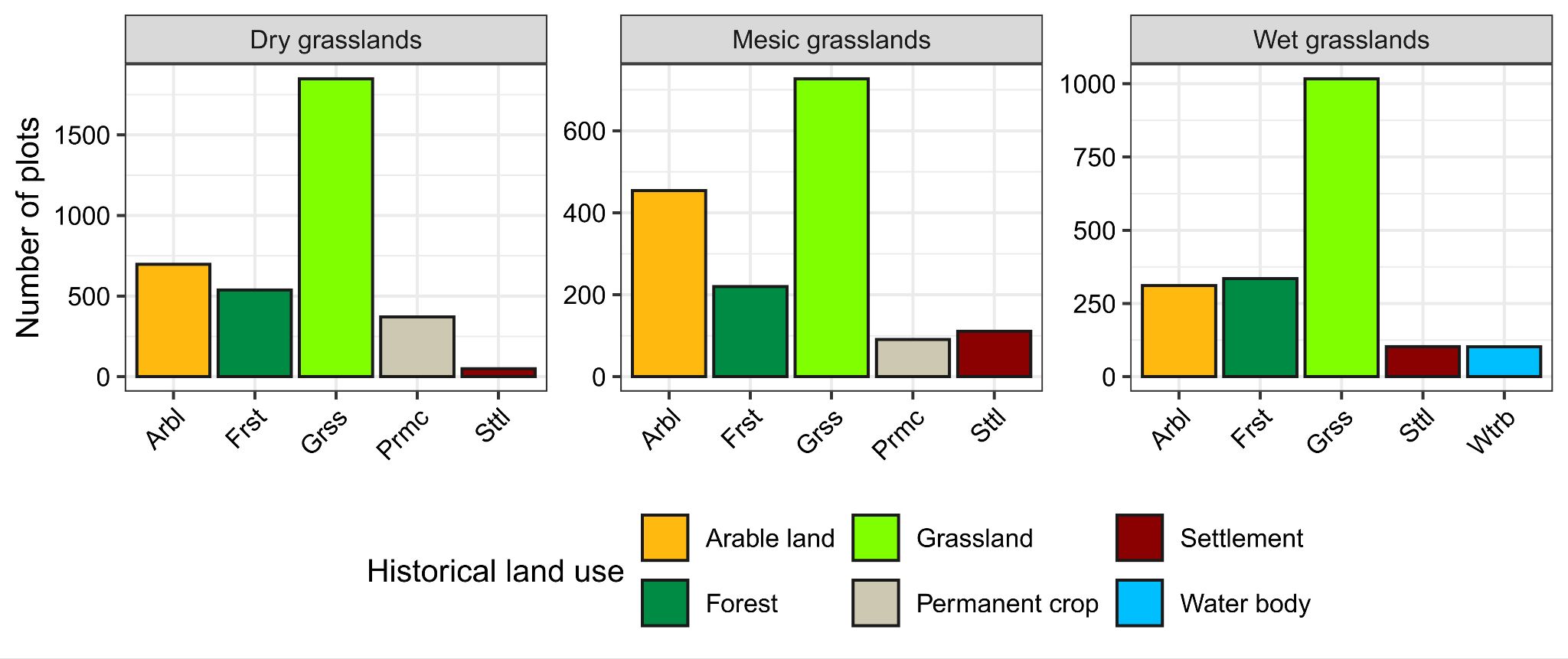
**


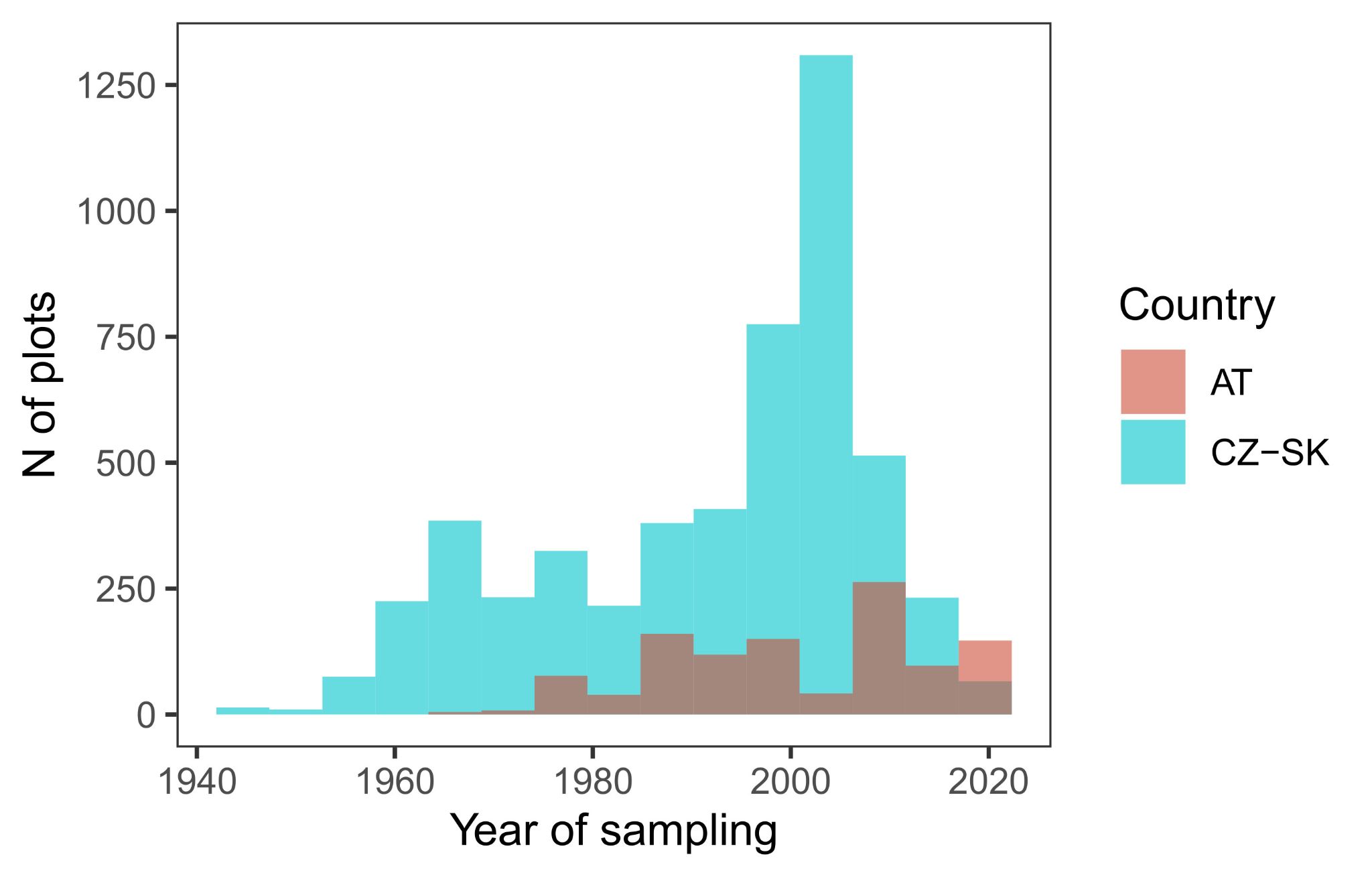
**Figure S1.1:** Number of vegetation plots included in the analysis for each habitat type categorized by historical land-use category.

**Figure S1.2:** Distribution of sampling year across the two country units (Austria and Czech Republic + Slovakia). The graph only includes plots with known sampling year (6,274 out of the 6,975 plots included in the analysis).

**Table S1.1:** List of most common EUNIS level-3 habitat types present in the study area. We selected these habitats based on our expertise and maps of habitat distributions based on the European Vegetation Archive (EVA) (Chytrý et al. 2020*; maps available at <https://floraveg.eu/>).

| **EUNIS habitat (level 3)** | **Habitat name** |
| --- | --- |
| Q11 | Raised bog |
| Q22 | Poor fen |
| Q24 | Intermediate fen and soft-water spring mire |
| Q25 | Non-calcareous quaking mire |
| Q42 | Extremely rich moss-sedge fen |
| Q51 | Tall-helophyte bed |
| Q52 | Small-helophyte bed |
| Q53 | Tall-sedge bed |
| Q54 | Inland saline or brackish helophyte bed |
| R12 | Cryptogam- and annual-dominated vegetation on siliceous rock outcrops |
| R13 | Cryptogam- and annual-dominated vegetation on calcareous and ultramafic rock outcrops |
| R16 | Perennial rocky grassland of Central and South-Eastern Europe |
| R1A | Semi-dry perennial calcareous grassland (meadow steppe) |
| R1B | Continental dry grassland (true steppe) |
| R1M | Lowland to montane, dry to mesic grassland usually dominated by Nardus stricta |
| R1P | Oceanic to subcontinental inland sand grassland on dry acid and neutral soils |
| R1Q | Inland sanddrift and dune with siliceous grassland |
| R21 | Mesic permanent pasture of lowlands and mountains |
| R22 | Low and medium altitude hay meadow |
| R23 | Mountain hay meadow |
| R35 | Moist or wet mesotrophic to eutrophic hay meadow |
| R36 | Moist or wet mesotrophic to eutrophic pasture |
| R37 | Temperate and boreal moist or wet oligotrophic grassland |
| R51 | Thermophilous forest fringe of base-rich soils |
| R55 | Lowland moist or wet tall-herb and fern fringe |
| R57 | Herbaceous forest clearing vegetation |
| R63 | Temperate inland salt marsh |
| S32 | Temperate Rubus scrub |
| S35 | Temperate and submediterranean thorn scrub |
| S36 | Low steppic scrub |
| S42 | Dry heath |
| S91 | Temperate riparian scrub |
| S92 | Salix fen scrub |
| T11 | Temperate Salix and Populus riparian forest |
| T12 | Alnus glutinosa-Alnus incana forest on riparian and mineral soils |
| T13 | Temperate hardwood riparian forest |
| T15 | Broadleaved swamp forest on non-acid peat |
| T16 | Broadleaved mire forest on acid peat |
| T17 | Fagus forest on non-acid soils |
| T18 | Fagus forest on acid soils |
| T19 | Temperate and submediterranean thermophilous deciduous forest |
| T1B | Acidophilous Quercus forest |
| T1E | Carpinus and Quercus mesic deciduous forest |
| T1F | Ravine forest |
| T31 | Temperate mountain Picea forest |
| T32 | Temperate mountain Abies forest |
| T35 | Temperate continental Pinus sylvestris forest |
| T3J | Pinus and Larix mire forest |
| T3K | Picea mire forest |
| V11 | Intensive unmixed crops |
| V13 | Arable land with unmixed crops grown by low-intensity agricultural methods |
| V15 | Bare tilled, fallow or recently abandoned arable land |
| V34 | Trampled xeric grassland with annuals |
| V35 | Trampled mesophilous grassland with annuals |
| V37 | Annual anthropogenic herbaceous vegetation |
| V38 | Dry perennial anthropogenic herbaceous vegetation |
| V39 | Mesic perennial anthropogenic herbaceous vegetation |

**Level-2 EUNIS habitat names**: Q1= "Raised and blanket bogs"; Q2= "Valley mires, poor fens and transition mires"; Q4 = "Base-rich fens and calcareous spring mires"; Q5 = "Helophyte beds"; R1 = "Dry grasslands"; R2 = "Mesic grasslands"; R3 = "Seasonally wet and wet grasslands"; R5 = "Woodland fringes and clearings and tall forb stands"; R6 = "Inland salt steppes and salt marshes"; S3 = "Temperate and Mediterranean-montane scrub"; S4 = "Temperate heathland"; S9 = "Riverine and fen scrub"; T1 = "Broadleaved deciduous forests"; T3 = "Coniferous forests"; V1 = "Arable land and market gardens"; V3 = "Artificial grasslands and herb-dominated habitats"

* **Reference**:

Chytrý, M., Tichý, L., Hennekens, S. M., Knollová, I., Janssen, J. A., Rodwell, J. S., ... & Schaminée, J. H. (2020). EUNIS Habitat Classification: Expert system, characteristic species combinations and distribution maps of European habitats. *Applied Vegetation Science*, *23*(4), 648-675. doi: <https://doi.org/10.1111/avsc.12519>

**
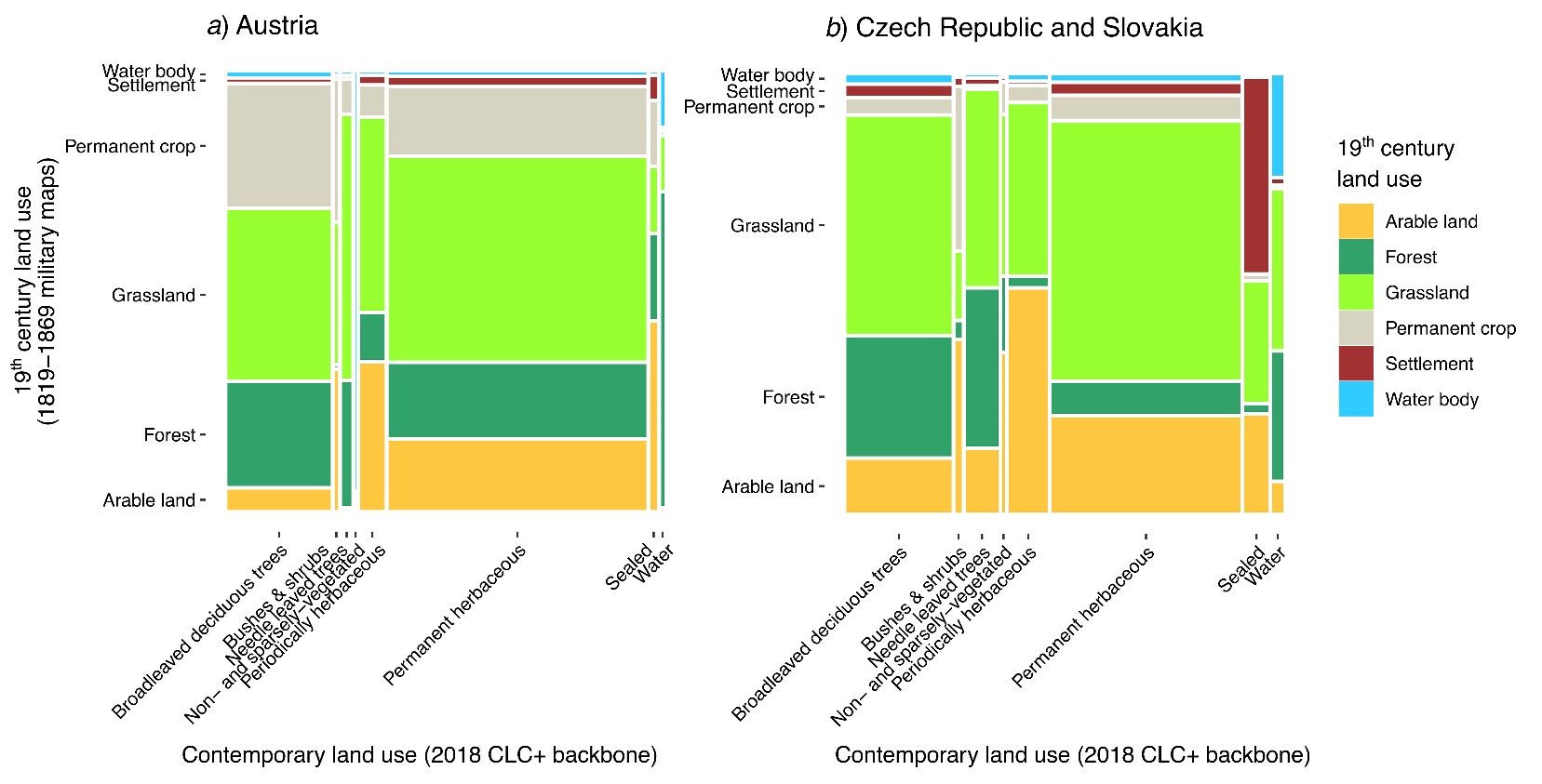
Figure S1.3:** Mosaic plots showing the conditional distributions in a contingency table (Table S1.2) of historical land use (1819-1869) vs. contemporary land cover (extracted using 10 m resolution CLC+ land cover for the year 2018) across the plots in a) Austria and b) Czech Republic and Slovakia.

**Table S1.2:** Contingency table of historical land use (1819-1869) vs. contemporary land use (extracted using 10 m resolution CLC+ land cover for the year 2018) across all 6,975 plots analyzed.

|  | | **CLC+Backbone (2018)** | | | | | | | | |
| --- | --- | --- | --- | --- | --- | --- | --- | --- | --- | --- |
|  |  | Broadleaved deciduous trees | | Bushes & shrubs | Needle leaved trees | Non- and sparsely-vegetated | Periodically herbaceous | Permanent herbaceous | Sealed | Water |
| **Historical land use (1819-69)** | Arable land | 200 | 37 | | 68 | 11 | 316 | 734 | 86 | 10 |
|  | Forest | 499 | 3 | | 179 | 5 | 18 | 330 | 9 | 50 |
|  | Grassland | 898 | 16 | | 230 | 11 | 255 | 2,028 | 98 | 56 |
|  | Permanent crop | 137 | 35 | | 2 | 2 | 22 | 259 | 5 | 0 |
|  | Settlement | 35 | 1 | | 3 | 0 | 1 | 72 | 155 | 1 |
|  | Water body | 26 | 0 | | 1 | 1 | 5 | 33 | 0 | 36 |

# **Appendix S2: Supplementary information for species richness and composition analyses**

**
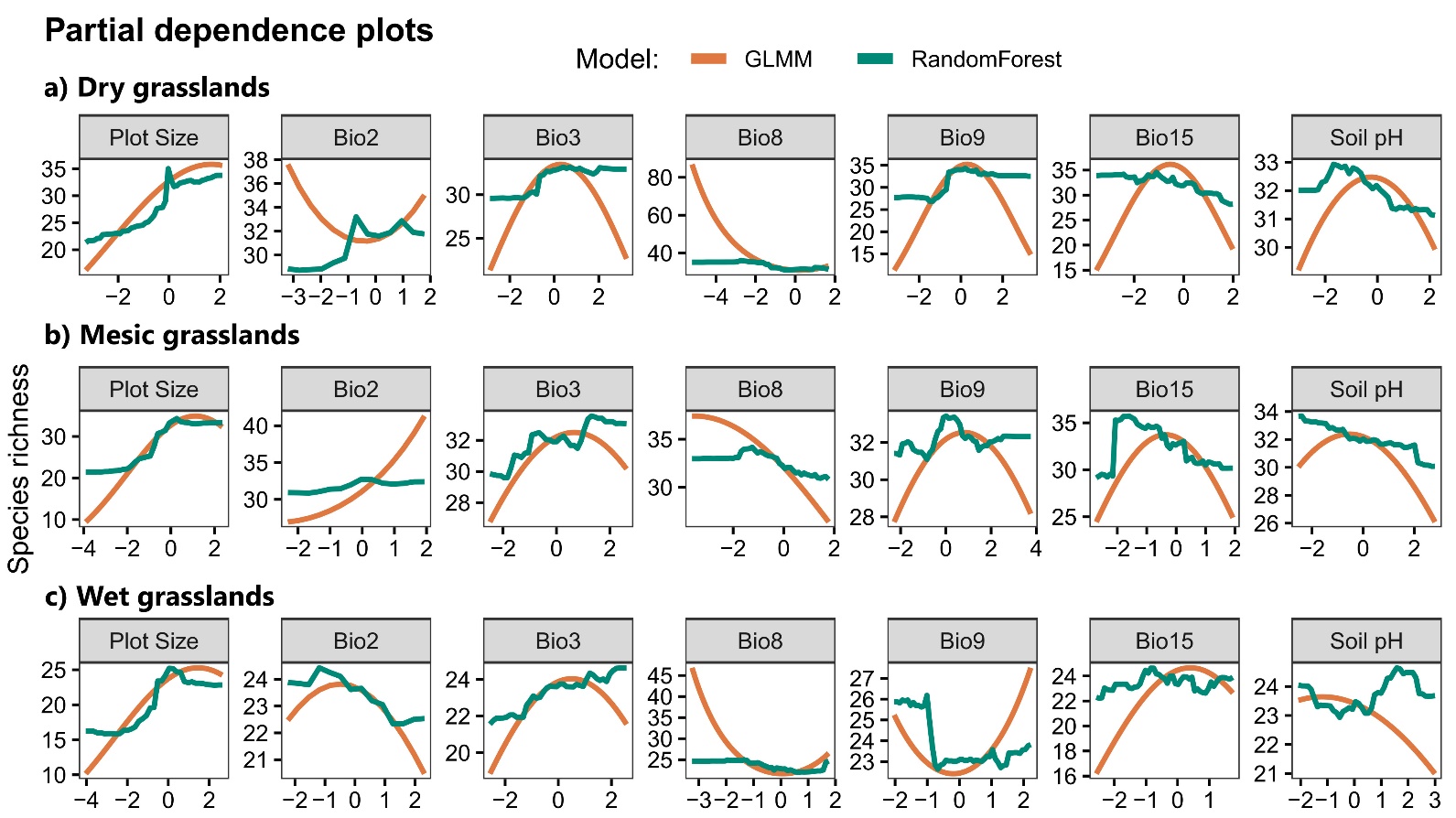
**

**Figure S2.1:** Panel matrix showing partial dependence effect of a single predictor continuous variable on species richness (columns) from generalized linear mixed models (GLMMs) and Random Forest models obtained for each habitat type separately (rows).


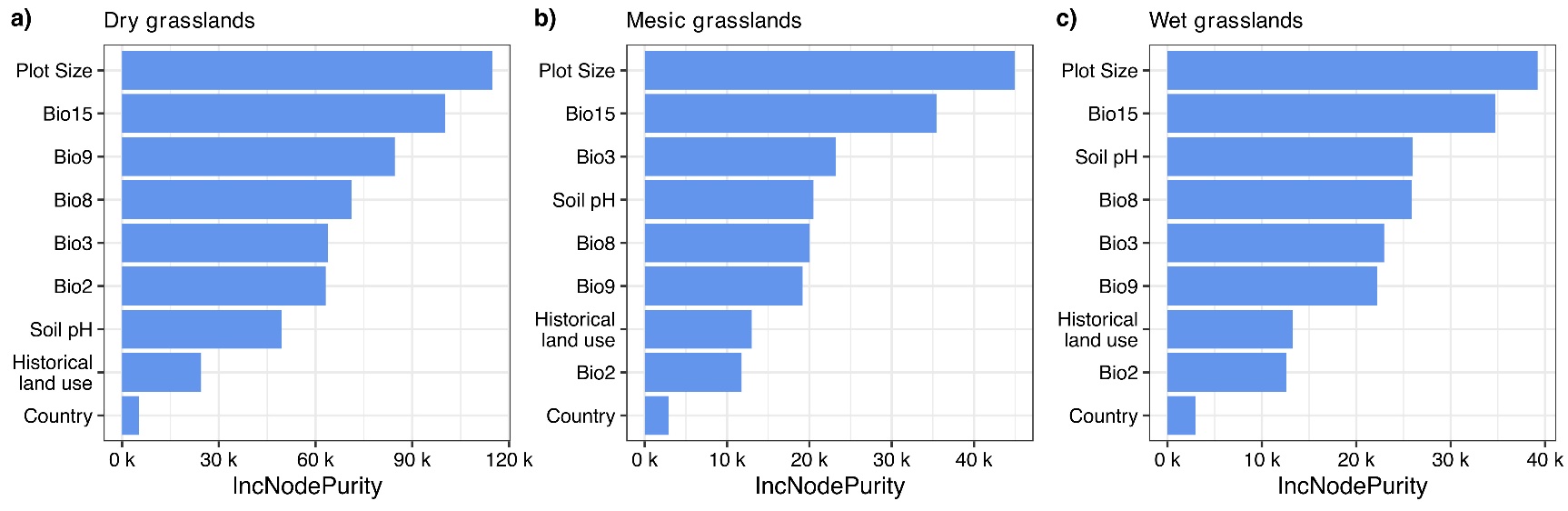


**Figure S2.3:** Variable importance (increase in node purity) for the different predictors considered in Random Forest models within each habitat separately.

**Table S2.1**: Results of PERMANOVA obtained across the three habitat types linking species composition to different prediction terms: historical land use; mean diurnal air temperature range ('bio2'); isothermality ('bio3'); mean daily mean air temperatures of the wettest quarter ('bio8'); mean daily mean air temperatures of the driest quarter ('bio9'); precipitation seasonality ('bio15'); soil pH; and country (Austria vs. Czech Republic and Slovakia). The table reports degrees of freedom, sum of suqares, R^2^ values, F test values and associated p-values (Pr) estimated over 100 permutations.

| **Habitat type** | **Term** | **Df** | **SumOfSqs** | **R^2^** | **F** | **Pr(>F)** |
| --- | --- | --- | --- | --- | --- | --- |
| **Dry grasslands** | Hist. Land Use | 4 | 0.019 | 0.019 | 19.59 | > 0.01 |
|  | Bio15 | 1 | 0.044 | 0.044 | 178.28 | > 0.01 |
|  | Bio2 | 1 | 0.016 | 0.016 | 64.93 | > 0.01 |
|  | Bio3 | 1 | 0.014 | 0.014 | 55.31 | > 0.01 |
|  | Bio8 | 1 | 0.002 | 0.002 | 8.11 | > 0.01 |
|  | Bio9 | 1 | 0.017 | 0.017 | 67.14 | > 0.01 |
|  | soil pH | 1 | 0.004 | 0.004 | 14.44 | > 0.01 |
|  | country | 1 | 0.019 | 0.019 | 76.52 | > 0.01 |
|  | Residual | 3,492 | 0.861 | 0.865 |  |  |
|  | Total | 3,503 | 0.994 | 1.000 |  |  |
| **Mesic grasslands** | Hist. Land Use | 4 | 0.037 | 0.064 | 32.41 | > 0.01 |
|  | Bio15 | 1 | 0.006 | 0.010 | 21.07 | > 0.01 |
|  | Bio2 | 1 | 0.043 | 0.075 | 151.76 | > 0.01 |
|  | Bio3 | 1 | 0.022 | 0.038 | 77.02 | > 0.01 |
|  | Bio8 | 1 | 0.001 | 0.002 | 4.83 | > 0.01 |
|  | Bio9 | 1 | 0.004 | 0.007 | 14.10 | > 0.01 |
|  | soil pH | 1 | 0.004 | 0.007 | 15.16 | > 0.01 |
|  | country | 1 | 0.005 | 0.010 | 19.26 | > 0.01 |
|  | Residual | 1,591 | 0.449 | 0.786 |  |  |
|  | Total | 1,602 | 0.571 | 1.000 |  |  |
| **Wet grasslands** | Hist. Land Use | 4 | 0.013 | 0.026 | 14.37 | > 0.01 |
|  | Bio15 | 1 | 0.004 | 0.007 | 15.99 | > 0.01 |
|  | Bio2 | 1 | 0.036 | 0.072 | 160.68 | > 0.01 |
|  | Bio3 | 1 | 0.021 | 0.042 | 93.81 | > 0.01 |
|  | Bio8 | 1 | 0.002 | 0.005 | 11.03 | > 0.01 |
|  | Bio9 | 1 | 0.001 | 0.001 | 2.65 | 0.03 |
|  | soil pH | 1 | 0.004 | 0.007 | 15.69 | > 0.01 |
|  | country | 1 | 0.001 | 0.003 | 6.58 | > 0.01 |
|  | Residual | 1,856 | 0.418 | 0.836 |  |  |
|  | Total | 1,867 | 0.500 | 1.000 |  |  |

# **Appendix S3: Supplementary information for species indicator analysis**

Ordination analysis on the indicator values

We restricted our analysis on indicator values to the first four orthogonal axes which explained more than 80% of the total cumulative variation of indicator values (following 'varimax' rotation) (Figure S3.1b). The first axis explained 26% of the variation and was positively correlated with disturbance frequency, mowing frequency, and light. This axis spans a gradient from species associated with unmown, shaded vegetation (e.g., forest understories) (e.g., *Anemone nemorosa*) to species tolerating frequently disturbed and light-exposed conditions (e.g., *Crepis biennis*, *Cynosurus cristatus*). The second axis explained 19% of the variation and was positively correlated with higher disturbance severity and soil disturbance. This axis spans from species typical of moderately disturbed habitats - like wetland vegetation (e.g., *Carex davalliana*, *Eriophorum angustifolium*) to species of severely disturbed vegetation such as arable land (e.g., *Chenopodium album*, *Veronica triphyllos*). The third axis explained 20% of the variation and was positively correlated with soil nutrients and moisture, while being negatively correlated with grazing pressure. This axis contrasted species found in dry, nutrient-poor habitats like rocky grasslands and rock outcrops (e.g., *Fumana procumbens*, *Gagea bohemica*) to species typical of wetlands with less severe or no grazing (e.g., *Glyceria maxima*, *Lycopus europaeus*). The fourth axis explained 16% of the variation and was positively correlated with higher soil reaction (i.e., lower soil acidity) and temperature. This axis captured a gradient from species growing in more acidic and colder sites (e.g., *Calluna vulgaris*, *Nardus stricta*) to more alkaline and warmer sites (e.g., *Cytisus austriacus*, *Iris pumila*).

# **
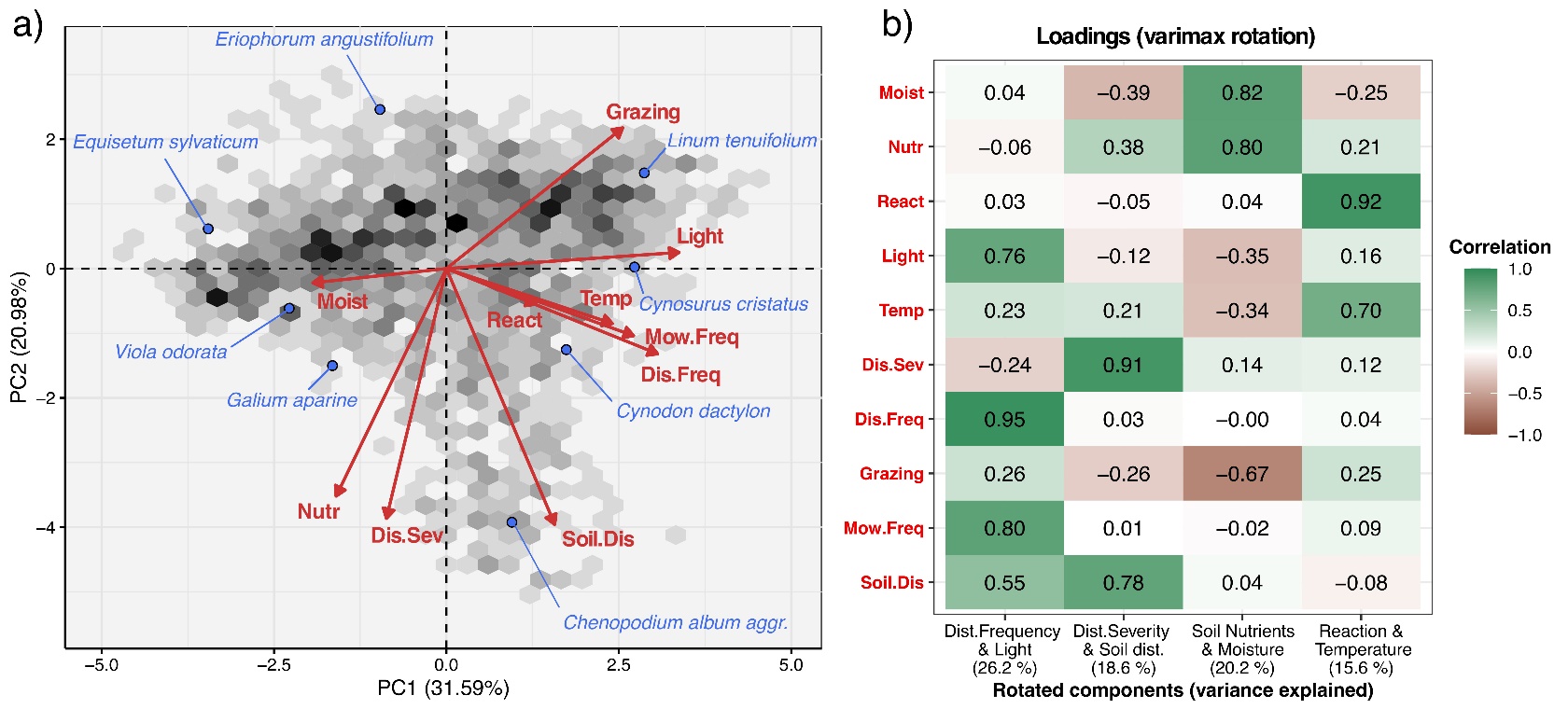
 Figure S3.1**: Biplot of the principal component analysis (PCA) conducted on ecological (= Ellenberg-like) and disturbance indicator values for the 1,461 selected species present in the study area (= panel *a*). Variables in the PCA are disturbance frequency ("Dis.Freq"), disturbance severity ("Dis.Seve"), mowing frequency ("Mow.Freq"), grazing pressure ("Grazing"), soil disturbance (“Soil.Dis”), and ecological optima for light ("Light"), soil moisture ("Moist"), soil nutrients ("Nutr"), soil reaction ("React"), and temperature ("Temp"). The hexagonal tessellation summarizes species positioning, with darker color values corresponding to higher species counts. Examples of species with contrasting ecological strategies are labeled in italics. Panel *b* displays the Pearson correlation coefficient between indicator values and varimax-rotated components used as response traits in the analysis.


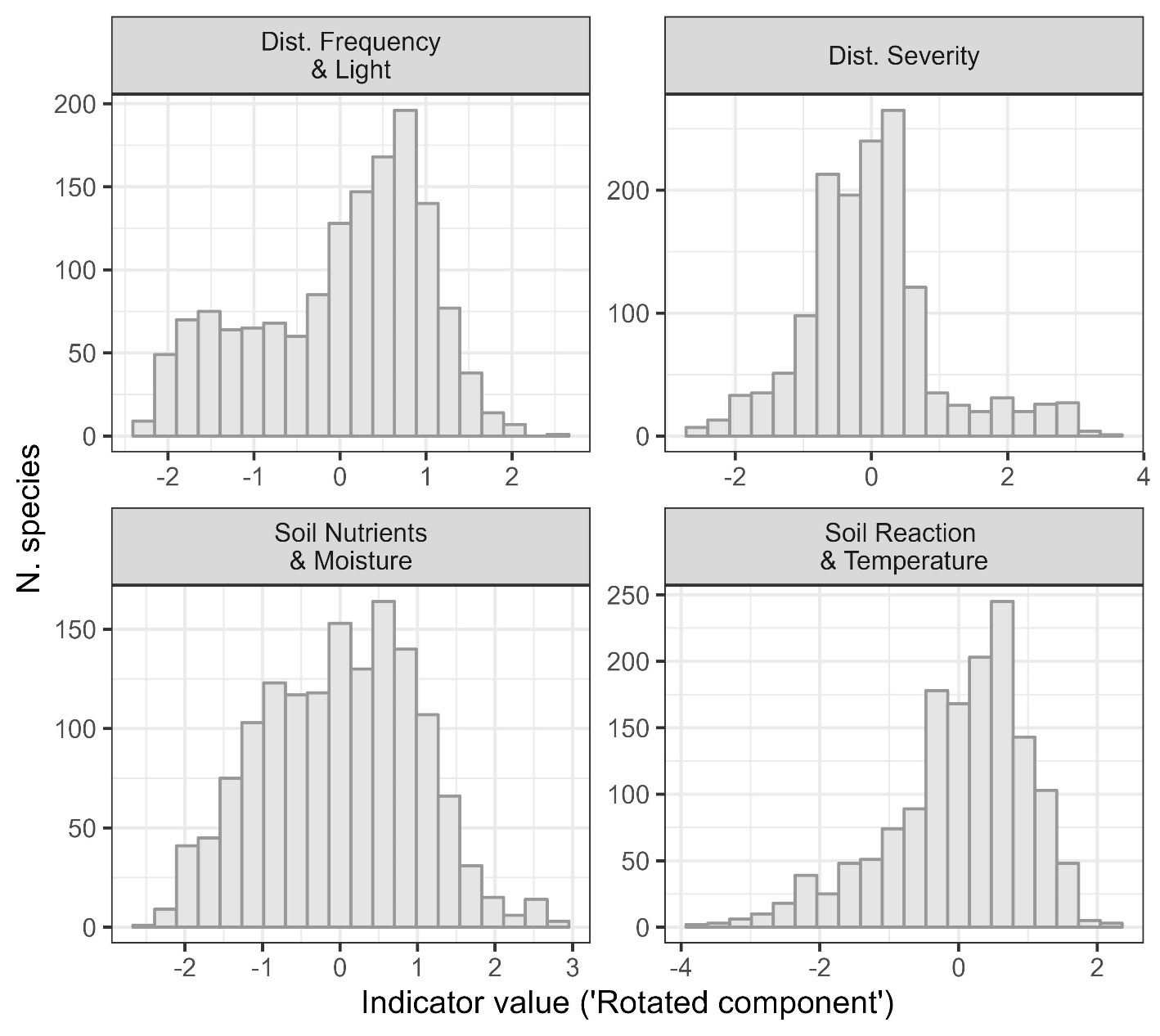
**Figure S3.2**: Distribution of the varimax-rotated components used as response traits in the analysis obtained from principal component analysis (PCA) conducted on ecological (= Ellenberg-like) and disturbance indicator values for the 1,498 selected species selected in the study area (see Figure S3.1).


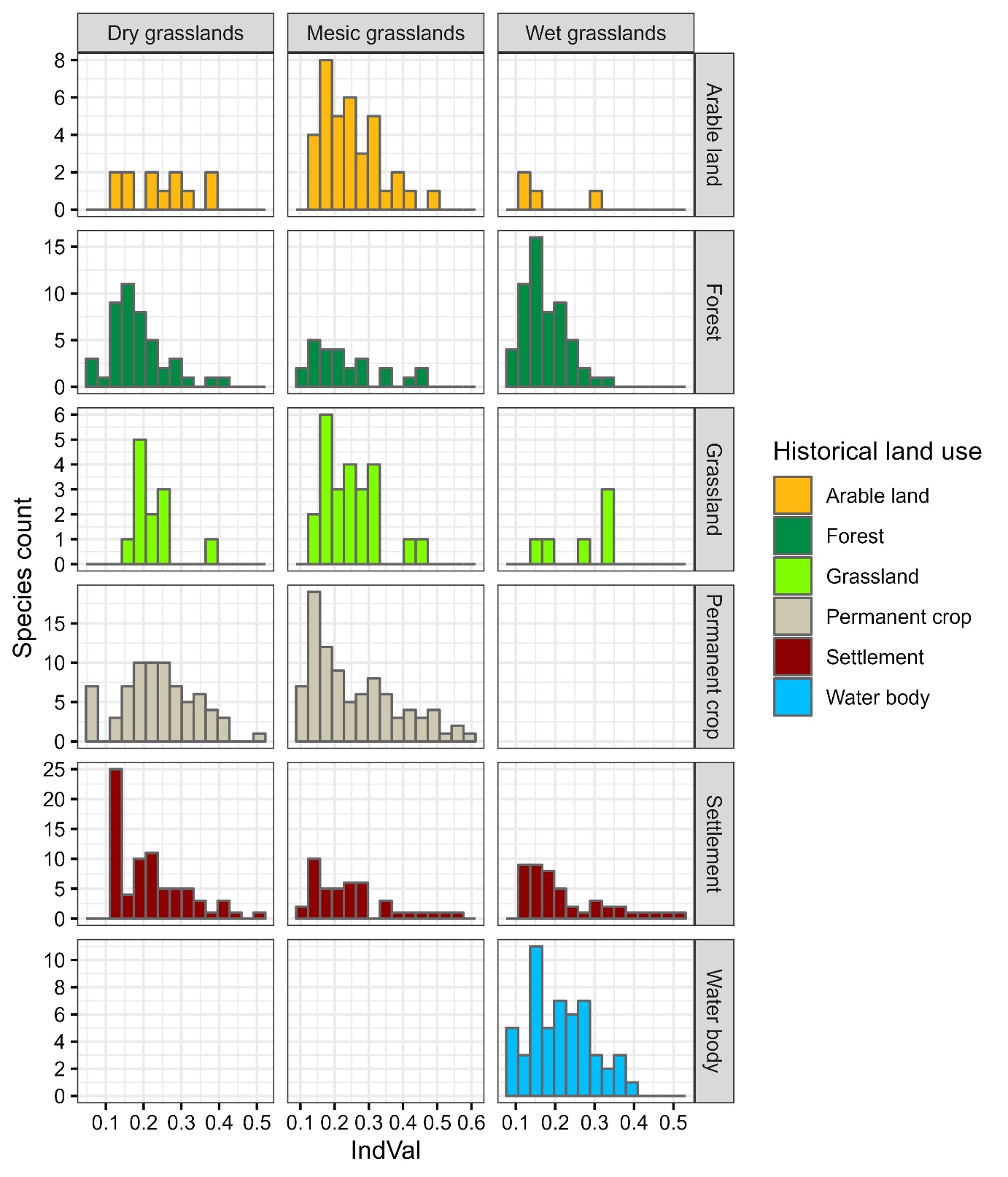
**Figure S3.3**: Count of species (y-axis) with significant (p-val < 0.05) IndVal statistic (x-axis) (492 individual species over 1,498 species considered in total) for each historical land-use category measured separately in each of the three habitat types (dry, mesic, and wet grasslands). We did not consider the ‘water body’ category for dry and mesic grasslands, and ‘permanent crop’ for wet grasslands because of too few plots available in such categories (see Materials and Methods).

**Table S3.1**: Top five species with the highest and significant (p-val < 0.05) Indicator Value (IndVal) statistic across the entire dataset. Here, historical land use and habitat type were combined as grouping variables. Thus, species were assigned to each group based on their frequency of occurrence in a specific historical land-use and habitat type combination. The table reports the p-value of IndVal and the number of plots where each species occurs within each habitat type. See the online data repository reporting complete results (.csv files) of indicator species analysis across the entire data set (`IndVal.all.habitats.csv`) and for each habitat type separately (`IndVal.separate.habitats.csv`). The latter was used for statistical comparisons with ecological preferences (see Table 1; Figure 4; Figure S3.3).

| Historical land use | Habitat | Species (top 5) | IndVal | p-val | Number of plots |
| --- | --- | --- | --- | --- | --- |
| Arable land | Dry grasslands | *Asperula cynanchica* | 0.35 | 0.001 | 1341 |
|  |  | *Brachypodium pinnatum* | 0.304 | 0.001 | 1275 |
|  |  | *Pimpinella saxifraga* | 0.285 | 0.001 | 1196 |
|  |  | *Galium verum* | 0.283 | 0.001 | 1416 |
|  |  | *Thymus* *odoratissimus* aggr. | 0.279 | 0.001 | 822 |
|  | Mesic grasslands | *Plantago lanceolata* | 0.421 | 0.001 | 1123 |
|  |  | *Lotus corniculatus* | 0.35 | 0.001 | 619 |
|  |  | *Agrostis capillaris* | 0.342 | 0.001 | 508 |
|  |  | *Daucus carota* | 0.335 | 0.001 | 330 |
|  |  | *Leontodon hispidus* | 0.329 | 0.001 | 553 |
|  | Wet grasslands | *Caltha palustris* | 0.315 | 0.001 | 586 |
|  |  | *Scirpus sylvaticus* | 0.296 | 0.001 | 532 |
|  |  | *Angelica sylvestris* | 0.29 | 0.001 | 563 |
|  |  | *Ranunculus* *auricomus* aggr. | 0.269 | 0.001 | 483 |
|  |  | *Cardamine pratensis* | 0.244 | 0.001 | 458 |
| Forest | Dry grasslands | *Teucrium chamaedrys* | 0.414 | 0.001 | 1023 |
|  |  | *Euphorbia cyparissias* | 0.358 | 0.001 | 1582 |
|  |  | *Vincetoxicum hirundinaria* | 0.322 | 0.001 | 453 |
|  |  | *Origanum vulgare* | 0.291 | 0.001 | 343 |
|  |  | *Verbascum chaixii* | 0.269 | 0.001 | 308 |
|  | Mesic grasslands | *Dactylis glomerata* | 0.407 | 0.001 | 1104 |
|  |  | *Poa* *pratensis* aggr. | 0.373 | 0.001 | 1073 |
|  |  | *Ranunculus* *acris* aggr. | 0.331 | 0.001 | 793 |
|  |  | *Leucanthemum* *vulgare* aggr. | 0.3 | 0.001 | 542 |
|  |  | *Anthoxanthum* *odoratum* aggr. | 0.29 | 0.002 | 646 |
|  | Wet grasslands | *Myosotis scorpioides* aggr. | 0.318 | 0.001 | 610 |
|  |  | *Impatiens noli-tangere* | 0.299 | 0.001 | 88 |
|  |  | *Chaerophyllum hirsutum* | 0.261 | 0.001 | 142 |
|  |  | *Carex remota* | 0.257 | 0.001 | 31 |
|  |  | *Ranunculus repens* | 0.256 | 0.001 | 631 |
| Grassland | Dry grasslands | *Potentilla incana* | 0.383 | 0.001 | 1316 |
|  |  | *Festuca valesiaca* aggr. | 0.292 | 0.001 | 896 |
|  |  | *Carex humilis* | 0.28 | 0.001 | 864 |
|  |  | *Thymus praecox* | 0.275 | 0.001 | 674 |
|  |  | *Sanguisorba minor* aggr. | 0.273 | 0.001 | 1147 |
|  | Mesic grasslands | *Festuca rubra* aggr. | 0.419 | 0.001 | 870 |
|  |  | *Veronica chamaedrys* aggr. | 0.354 | 0.001 | 901 |
|  |  | *Rumex acetosa* | 0.354 | 0.001 | 813 |
|  |  | *Cerastium* *fontanum* subsp. *vulgare* | 0.284 | 0.001 | 704 |
|  |  | *Campanula* *patula* aggr. | 0.277 | 0.001 | 459 |
|  | Wet grasslands | *Carex nigra* | 0.315 | 0.001 | 498 |
|  |  | *Filipendula ulmaria* | 0.305 | 0.001 | 606 |
|  |  | *Cirsium palustre* | 0.275 | 0.001 | 610 |
|  |  | *Galium* *palustre* aggr. | 0.264 | 0.001 | 559 |
|  |  | *Silene flos-cuculi* | 0.253 | 0.001 | 550 |
| Permanent crop | Dry grasslands | *Centaurea scabiosa* | 0.425 | 0.001 | 1256 |
|  |  | *Peucedanum cervaria* | 0.411 | 0.001 | 514 |
|  |  | *Seseli libanotis* | 0.391 | 0.001 | 181 |
|  |  | *Galatella linosyris* | 0.372 | 0.001 | 437 |
|  |  | *Aster amellus* | 0.372 | 0.001 | 392 |
|  | Mesic grasslands | *Arrhenatherum elatius* | 0.544 | 0.001 | 904 |
|  |  | *Tragopogon pratensis* | 0.471 | 0.001 | 225 |
|  |  | *Trifolium pratense* | 0.469 | 0.001 | 789 |
|  |  | *Centaurea jacea* | 0.441 | 0.001 | 538 |
|  |  | *Trisetum flavescens* | 0.43 | 0.001 | 577 |
| Settlement | Dry grasslands | *Poa compressa* | 0.483 | 0.001 | 143 |
|  |  | *Arenaria serpyllifolia* | 0.398 | 0.001 | 595 |
|  |  | *Sisymbrium loeselii* | 0.397 | 0.001 | 9 |
|  |  | *Berteroa incana* | 0.382 | 0.001 | 154 |
|  |  | *Sedum album* | 0.368 | 0.001 | 461 |
|  | Mesic grasslands | *Taraxacum* sect. *Taraxacum* | 0.521 | 0.001 | 921 |
|  |  | *Pastinaca sativa* | 0.486 | 0.001 | 247 |
|  |  | *Trifolium repens* | 0.404 | 0.001 | 660 |
|  |  | *Crepis biennis* | 0.387 | 0.001 | 243 |
|  |  | *Convolvulus arvensis* | 0.37 | 0.001 | 219 |
|  | Wet grasslands | *Plantago major* | 0.441 | 0.001 | 293 |
|  |  | *Argentina anserina* | 0.438 | 0.001 | 251 |
|  |  | *Lolium perenne* | 0.403 | 0.001 | 129 |
|  |  | *Ochlopoa annua* | 0.365 | 0.001 | 129 |
|  |  | *Polygonum aviculare* aggr. | 0.36 | 0.001 | 117 |
| Water body | Wet grasslands | *Lysimachia vulgaris* | 0.388 | 0.001 | 559 |
|  |  | *Lythrum salicaria* | 0.358 | 0.001 | 242 |
|  |  | *Carex panicea* | 0.35 | 0.001 | 473 |
|  |  | *Agrostis stolonifera* | 0.339 | 0.001 | 367 |
|  |  | *Molinia caerulea* aggr. | 0.324 | 0.001 | 321 |
